# The context-dependent role of the dsRNA response in linking A-to-I editing and ADAR to normal hematopoiesis and leukemia

**DOI:** 10.3389/fcell.2026.1799320

**Published:** 2026-05-25

**Authors:** Xiaowei Li, Jiayue Xu, Jia Yu

**Affiliations:** 1 4+4 Medical Doctor Program, Chinese Academy of Medical Sciences & Peking Union Medical College, Beijing, China; 2 Institute of Basic Medical Sciences Chinese Academy of Medical Sciences, School of Basic Medicine Peking Union Medical College, Beijing, China

**Keywords:** ADAR, dsRNA sensing, hematopoiesis, leukemia, RNA editing

## Abstract

Adenosine-to-inosine (A-to-I) RNA editing, catalyzed by adenosine deaminase acting on RNA (ADAR), contributes to cellular RNA homeostasis. By recoding protein, inducing alternative splicing and regulating non-coding RNAs, ADAR-mediated editing influences diverse biological processes including development, homeostasis and pathogenesis. In particular, ADAR-mediated A-to-I editing prevents endogenous double-stranded RNAs from improperly activating pattern recognition receptors and downstream innate immune response (dsRNA response), thereby modulating immune homeostasis in a context-dependent manner. Compelling evidence from human studies and animal models implicates ADAR-mediated A-to-I editing in key hematological processes including embryonic and adult hematopoiesis. Notably, dysregulated ADAR functions and aberrant A-to-I editing are increasingly associated with leukemia pathogenesis and progression, thereby exihibiting novel clinical potentials. Given the intimate crosstalk between the hematological and immune systems, it is likely that ADAR’s immunosuppressive function mediates these pleiotropic effects of hematological A-to-I editing. In this review, we synthesize current knowledge on A-to-I editing and ADAR in normal hematopoiesis and leukemia. We then reinterpret these findings from an immunological perspective. We highlight the context-dependence of the ADAR1-dsRNA response axis, and characterize it from three perspectives: 1) the shift in axis requirement from normal to malignant states; 2) the involvement of distinct dsRNA sensor(s); 3) the relative hierarchy between editing-dependent mechanism and editing-independent mechanism. Specifically, we propose that the axis is generally indispensable for normal hematopoiesis, whereas its requirement may exhibit intra-leukemia and inter-leukemia heterogeneity. Based on this heterogeneity, we further discuss the diverse translational potentials of this axis, including therapeutic strategies, resistance biomarkers and prognostic predictors. Finally, we identify key theoretical gaps and methodological limitations in exploring this axis, hoping to inspire novel research directions and advance clinical management for leukemia.

## 1 Introduction

As one of the most common post-transcriptional modifications, RNA editing refers to changes in the nucleotide sequence of an RNA transcript, including insertion, deletion and base substitution. The most prevalent RNA editing type in metazoans is adenosine-to-inosine (A-to-I) editing, catalyzed by adenosine deaminases acting on RNA (ADARs) (Zhu et al., 2023). Upon binding to double-stranded RNA (dsRNA), ADARs convert adenosines (A) to inosines (I) through deamination. The resultant I is recognized as guanosine (G), which can destabilize base pairing and introduce A-to-G discrepancies (Weng et al., 2023). The vast majority of mammalian A-to-I events occur within repetitive sequences, such as human Alu elements, which readily form dsRNA structures.

The discovery of ADAR dates back to the 1980s, when it was first reported to unwind dsRNA via A-to-I editing in *Xenopus* oocytes and eggs (Bass and Weintraub, 1987; Rebagliati and Melton, 1987). A milestone was reached in 2000 with the identification of ADAR2-mediated recoding events in the transcript of a mammalian neurotransmitter receptor, pioneering the exploration of RNA editing (Higuchi et al., 2000). To date, ADAR has been reported to play crucial roles in development (Yang et al., 2021; Liang et al., 2024), homeostasis (Eisenberg and Levanon, 2018) and diseases (Song et al., 2022) across multiple systems, employing both editing-dependent and editing-independent mechanisms.

The hematological system, a finely tuned system, possesses unique features that make it particularly valuable for studying RNA editing. First, its fluidity and openness allow diseased elements to populate systemically. Although this increases the risk of systemic dissemination, it significantly improves the availability of pathological specimens, which not only facilitates clinical diagnosis, disease monitoring, and drug delivery but also simplifies the acquisition of primary cells and the construction of humanized mouse models. Second, the system exhibits remarkable plasticity due to the capacity of hematopoietic stem cells (HSCs) for self-renewal and multipotent differentiation, which renders it particularly dependent on precise regulation. RNA editing, representing a regulated layer of transcriptome plasticity that operates under immune surveillance constraints, contributes to this regulatory demand. Last but not least, the intrinsic link between hematology and immunity is critical. Since ADAR and A-to-I editing also regulate the immune responses, it is highly promising to explore their influences on hematology through immunological mechanisms.

To date, it is widely recognized that ADAR and A-to-I editing play an imperative role in both normal hematopoiesis and hematologic diseases. While other reviews have specifically addressed hematological RNA editing (Teoh et al., 2021; Pu et al., 2025), this review aims to elucidate the roles of ADAR and A-to-I editing in hematopoiesis and leukemia from an immunological perspective. We will first introduce the fundamentals of ADAR family and A-to-I editing, and then organize current findings in normal hematopoiesis and leukemia, respectively. We will conclude by discussing how the dsRNA response—a form of cellular immune response—mediates these functions.

## 2 The fundamentals of A-to-I editing and ADAR

### 2.1 ADAR family

**FIGURE 1:**
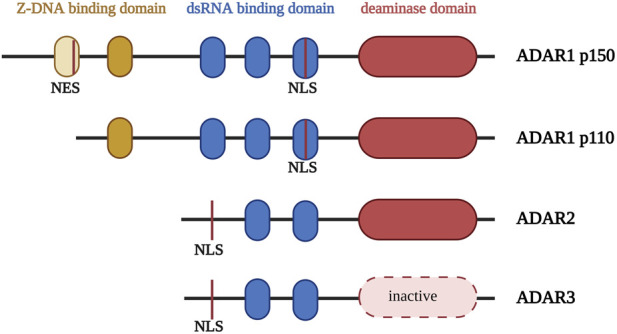
Domain arrangement of mammalian ADAR proteins. Each ADAR member possesses a C-terminal deaminase domain and dsRNA binding domains, accompanied by a nuclear localization signal (NLS). ADAR1 is uniquely characterized by Z-DNA binding domain(s), with the interferon-induced p150 isoform harboring two Z-DNA binding domains along with a nuclear export signal (NES). Created with www.biorender.com.

ADARs are highly conserved across vertebrates. In mammals, there are three ADAR family proteins with similar domain arrangement (Figure 1). For each protein, the deaminase domain is located at C-terminal, followed by several nucleic acid binding domains. ADAR1 possesses three dsRNA binding domains (dsRBDs) along with additional Z-DNA binding domains (ZBDs), while ADAR2 and ADAR3 contain only two dsRBDs (Zhang et al., 2024). ADAR1 expression is regulated by an interferon (IFN)-inducible alternative promoter, allowing the constitutive 110-kDa protein (ADAR1 p110/p110) to be partially replaced by an N-terminally extended 150-kDa isoform (ADAR1 p150/p150) upon viral infection (George and Samuel, 1999). The primary structural difference between two isoforms is the presence of an additional Z-DNA binding domain (Zα) in p150, which contains a nuclear export signal (NES) (Herbert et al., 1997).

ADARs differ in tissue distribution, subcellular localization, substrate preference and functional specialization due to detailed domain structures. In human, ADAR1 and ADAR2 are ubiquitously expressed, while ADAR3 expression is confined to the brain (Tan et al., 2017). ADAR1 p110, ADAR2 and ADAR3 are primarily localized in the nucleus. In contrast, ADAR1 p150 is predominantly cytoplasmic but can shuttle between the nucleus and cytoplasm (Datta et al., 2023). The primary substrates for ADARs are RNA duplexes formed by intramolecular base pairing of inversely oriented repetitive elements. ADAR1 mainly catalyzes editing in non-coding regions, leading to either single-site editing or clustered hyper-editing (Tan et al., 2017). ADAR2 is mostly reported to edit coding regions of central nervous system transcripts. ADAR3, despite containing a deaminase domain, lacks catalytic activity (Chen et al., 2000). It primarily modulates RNA editing by potentially competing for RNA binding sites, thereby antagonizing editing activities of ADAR1 and ADAR2 (Oakes et al., 2017).

### 2.2 Molecular and biological functions of RNA editing

**FIGURE 2:**
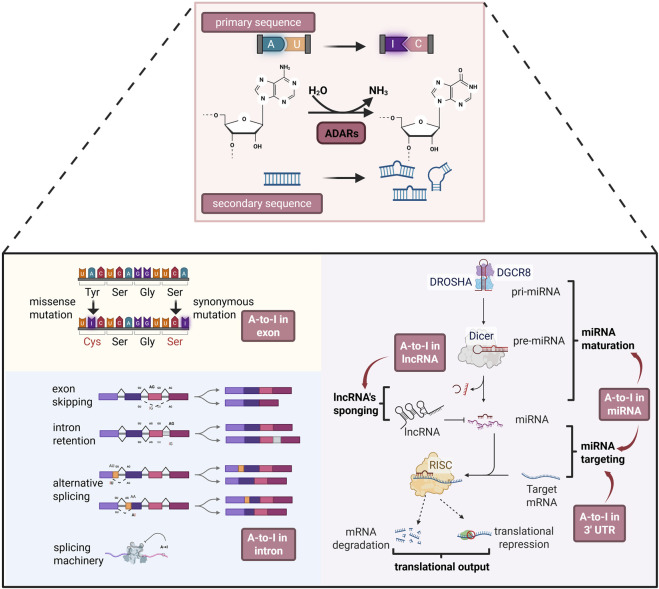
Molecular functions of A-to-I editing. A-to-I editing maintains RNA homeostasis through altering primary sequence and remodeling secondary structure. Its effects are largely determined by genomic locations: exonic editing causes protein recoding. Intronic editing affects splicing by modifying splicing sites or interfering with splicing machinery. 3′UTR editing influences mRNA stability and miRNA targeting. For non-coding RNAs, miRNA editing affects miRNA maturation and targeting, thereby regulating translational output. LncRNA editing also regulates translation indirectly by modulating its miRNA “sponge” activity. Created with www.biorender.com.

The molecular functions of RNA editing are determined by its genomic location (Figure 2). The principle is that editing directly alters the primary RNA sequence and can modify its secondary structure. Sequence changes affect genetic information flow and base-pairing properties, while structural alterations can disrupt inter-molecular interactions.

RNA editing in coding sequences, though relatively rare, can alter codons, causing protein mutation (Higuchi et al., 2000). The majority of RNA editing events occur in non-coding sequences. For introns, RNA editing primarily affects splicing. Splicing site (GU-AG) could be created and abolished via “AU > IU”/“AA>AI” and “AG > IG,” respectively, thereby causing alternative splicing, exon skipping or intron retention. Editing can also directly interact with splicing machinery (Goncharov et al., 2022). For untranslated regions (UTRs), RNA editing modulates mRNA metabolism and translation efficiency by influencing structural stability and altering microRNA (miRNA) binding sites (Zhang et al., 2024). RNA editing in non-coding RNAs (ncRNAs) is equally important. For example, it can interfere with miRNA maturation and alter miRNA target specificity (Liao et al., 2020). Also, RNA editing can generate or eliminate miRNA-binding sites on lncRNAs, modulating their miRNA “sponge” activity to regulate mRNA expression indirectly (Liao et al., 2020). Therefore, RNA editing is crucial for maintaining cellular RNA homeostasis, with its biological impacts being highly diverse and context-dependent.

By regulating RNA homeostasis, RNA editing participates in multiple signaling pathways, influencing cell proliferation, differentiation, apoptosis, and immune responses (Song et al., 2022). There has been substantial evidence supporting its role in suppressing innate immunity, especially the dsRNA response (see below).

Regarding editing enzymes, ADAR proteins have both editing-dependent and editing-independent functions. Dual mechanisms for ADAR1 have been reported in miRNA biosynthesis, as ADAR1 inhibits miRNA cleavage (catalyzed by DROSHA or DICER) through both pri-miRNA editing and binding competition (Yang et al., 2006; Kawahara et al., 2007). Interestingly, an ADAR1-DICER heterodimer promotes cleavage instead (Ota et al., 2013). Therefore, enzymes are not equal to RNA editing. ADAR’s editing and non-editing functions should be scrutinized carefully.

### 2.3 A-to-I editing and ADAR in the dsRNA response

To combat viruses, mammalian cells utilize a multi-layered dsRNA response involving cytosolic sensors (the retinoic acid-inducible gene I (RIG-I)-like receptors (RLRs), the protein kinase R (PKR), the oligoadenylate synthases (OAS) and the Z-DNA binding protein 1 (ZBP1) that trigger various antiviral effects from IFN signaling to cell death. As RLR members, melanoma differentiation-associated protein 5 (MDA5), RIG-I and laboratory of genetics and physiology 2 (LGP2) bind to dsRNAs cooperatively, forming filaments along dsRNAs, activating adaptor protein mitochondrial antiviral-signaling protein (MAVS) and inducing expression of type I/III IFN (hereinafter, “IFN” or “interferon” specifically refers to type I/III interferon) and pro-inflammatory cytokines. In contrast, dsRNA binding causes PKR dimerization and activation, leading to global translation shutdown and an integrated stress response (ISR). Functioning as a nucleotidyl transferase, OAS initiates non-specific RNA cleavage of ribonuclease L (RNase L) upon dsRNA binding. ZBP1, characterized by its Zα domains, binds to Z-nucleic acids specifically, recruits certain kinases and triggers programmed cell death (de Reuver and Maelfait, 2024). Notably, crosstalk across different sensing pathways complicates this system. For example, as interferon-stimulated genes (ISGs), PKR and OAS may be upregulated following MDA5 activation. In turn, PKR can augment MDA5-mediated signaling through directly interacting with MDA5 and stabilizing interferon transcripts. OAS can amplify dsRNA ligands for MDA5. Moreover, PKR and OAS can coordinate to regulate IFN signaling in a cell-type specific pattern (Chan and Jin, 2022). However, although different sensors have specific dsRNA preference and sensing mechanisms (de Reuver and Maelfait, 2024), the hierarchy of the dsRNA response remains undefined.

**FIGURE 3:**
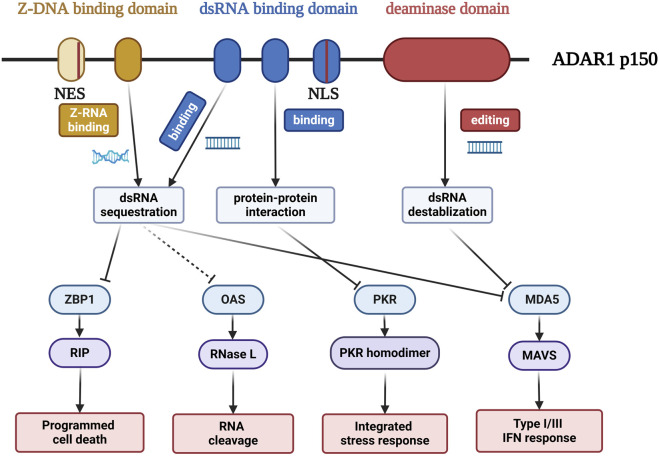
Regulation of ADAR1 p150 on dsRNA response. ADAR1 p150 suppresses MDA5-MAVS-IFN pathway through both editing-dependent dsRNA destabilization and binding-dependent dsRNA sequestration. It inhibits PKR sensing primarily by binding-dependent protein-protein interaction. For other dsRNA sensors, ADAR1 p150 inhibits OAS and ZBP1 sensing via dsRNA sequestration, with the Z-DNA binding activity specifically mediating ZBP1 regulation. Created with www.biorender.com.

The presence of endogenous dsRNAs–mostly originating from inversely oriented repetitive elements- necessitates precise distinction between self and non-self dsRNAs. ADARs’ substrate preference for dsRNAs and dsRNA-binding capability make them key regulators of the dsRNA response (Figure 3). Generally, to prevent endogenous dsRNAs from improperly activating this response, ADARs (primarily ADAR1) utilize both catalytic and binding activities: (i) editing-dependent alteration of dsRNA structure or identity, and (ii) binding-dependent mechanisms, including dsRNA sequestration and direct interference with sensors (Hu and Li, 2024). For simplicity, such regulation is hereinafter referred to as “the ADAR1-dsRNA response axis.” As discussed above, the cytoplasmic localization, unique Zα domain, and IFN inducibility determine the principal role of ADAR1 p150 in this axis (Pestal et al., 2015; Kim et al., 2021; Sun et al., 2025). ADAR1 p150 suppresses MDA5-mediated IFN response through both editing-dependent dsRNA destabilization and editing-independent dsRNA sequestration (Nakahama and Kawahara, 2023). Moreover, there might be a negative feedback loop maintaining immune homeostasis, given that IFN upregulates ADAR1 p150 and ADAR1 p150 in turn suppresses MDA5-MAVS-IFN signaling. ADAR1 p150 also inhibits PKR signaling through direct protein-protein interaction, which is exploited by both viruses and tumors (Hu et al., 2023). Regarding ADAR1’s suppression on OAS-RNase L pathway, it may primarily prevent cell death (Li et al., 2017). Furthermore, ADAR1 p150 directly antagonizes ZBP1-triggered programmed cell death, likely through competition for Z-RNA ligands (Hu et al., 2023).

In short, ADAR1 p150’s immunosuppression is a double-edged sword, which prevents self-directed immunopathology but also facilitates virus invasion and tumor immune escape. Nevertheless, it has been questioned to what extent the immune homeostasis depends on ADAR1, given that most ADAR1-targeted events, typically occurring within nuclear pre-mRNAs, exhibit low editing levels, and are normally spliced without interacting with cytoplasmic dsRNA sensors (Levanon et al., 2024). In other words, to what extent do ADAR1’s substrates overlap with immunogenic dsRNAs? Two models have been proposed to address this issue, respectively emphasizing the quality or the quantity of endogenous dsRNAs as the determinant of immunogenicity. The “quality” model acknowledges the limited overlap, but suggests that few ADAR1-targeted dsRNAs are truly competent to initiate the dsRNA response, thereby requiring ADAR1’ immunosuppression to protect against false activation (Levanon et al., 2024). Identifying critical characteristics of such “qualified” dsRNAs would bolster this model. The “quantity” model posits a balance between dsRNA burden and tolerance threshold, proposing that all or most dsRNAs contribute to immunogenicity, and sensors are improperly alarmed once the dsRNA load exceeds the activation threshold. Consequently, ADAR1’s activities, although individually minor, collectively reduce the overall dsRNA burden, thereby maintaining the balance (de Reuver and Maelfait, 2024; Levanon et al., 2024). The concept of “dsRNA tolerance threshold” here is intriguing, complicating ADAR1’s role in regulating dsRNA response.

### 2.4 Approaches to study A-to-I editing and ADAR

The important roles of A-to-I editing and ADARs in homeostasis and diseases highlight the necessity to advance RNA editing site (RES) identification methods and protein functional assays.

The core of A-to-I editing site identification is to pinpoint DNA-RNA (cDNA) differences and/or detect the resulting inosines. Current RES identification methods fall into three categories: sequencing-based methods, chemically-assisted methods and enzyme-assisted methods (Yang and Sakurai, 2025). The conventional sequencing-based methods compare nucleotide differences between genomic DNA and transcriptomic cDNA sequences obtained from low-throughput techniques (e.g., Sanger sequencing) or high-throughput techniques, in which inosines are indirectly detected as guanosines and must be carefully distinguished from genuine guanosines. Innovatively, long-read sequencing, directly detecting inosines via specific electrical signals, opens a new avenue for accurate RES identification (Bortoletto and Rosani, 2024). Chemically-assisted methods and enzyme-assisted methods utilize chemical compounds and enzymes to specifically react with and cleave at inosine sites, respectively, causing cDNA abnormalities that can be identified by sequencing. Although these two approaches represent direct ways to detect inosines, their efficiency is limited by specificity and sensitivity of the compound/enzyme, and experimental conditions. Therefore, all current RES detection methods are imperfect, and the development of sensitive, accurate, and high-throughput detection technologies is still on the way. Additionally, a growing number of RNA editing database (e.g., REDIportal (D'Addabbo et al., 2025) have been established, providing valuable resources for studying RNA editing.

The era of high-throughput sequencing brings both opportunities and challenges to sequencing-based RES identification. Arising from both biological and technical sources, false positives represent the toughest problem, including but not limited to genetic mutations, single-nucleotide polymorphisms (SNPs), other RNA modifications, sequencing errors and misalignment. To avoid them as much as possible, we recommend a workflow covering four steps: data preprocessing, candidate identification, computational filtering and experimental validation. Data preprocessing refers to standard pipeline for RNA-seq data processing, including quality control, trimming, mapping and BAM file preprocessing. A wise choice on sequencing technology can pave the way for high reliability of RES identification. While bulk RNA-seq remains the most commonly used technology here, one might propose that single-cell RNA-seq would be more powerful to reveal cell-specific editomes. However, the low RNA input during single-cell library construction compromises its feasibility in RES identification, since the following “predicting” requires high sequencing coverage (Karagianni et al., 2024). The low sequencing depth may be overcome by optimizing “preprocessing,” such as establishing a pseudo-bulk RNA-seq dataset for each cell type (Wu et al., 2023). Additionally, strand-specific sequencing data, which retain strand information, can improve accuracy by distinguishing genuine A-to-G mismatches from T-to-C ones in antisense transcripts. Candidate identification denotes finding single nucleotide variants (SNVs) between DNA and RNA. Inferior to matched DNA and RNA sequence comparison (i.e., both DNA-seq and RNA-seq are performed for the same sample), comparing RNA-seq data with the reference genome may falsely identify SNPs as RES candidates. Computational filtering aims to remove false positives by applying various parameters such as read coverage, mapping quality, RES frequency and position. And candidates generated from above inferior comparing method must undergo SNP exclusion (Bortoletto and Rosani, 2024). Experimental validation, the best solution on false positives, utilizes various complementary methods to confirm the filtered putative RESs, such as targeted amplicon sequencing, Sanger sequencing and functional assays linking specific sites to phenotypes.

Regarding ADARs, their structures and functions have been extensively studied using multiple tools. Here, we highlight mouse models combined with cellular and genetic approaches as they are central to investigating ADARs in hematology. Various mouse models have been developed with smart gene manipulation strategies to delineate molecule-specific and context-dependent functions of ADAR family. Genetically, both constitutive and conditional gene knockout (KO) models are available. Molecularly, the target includes specific protein (e.g., *Adar1* ^−/−^ or *Adar2* ^−/−^), specific isoform (e.g., *Adar1 p110* ^−/−^ or *Adar1 p150* ^−/−^) or specific domain. Specifically, ADAR1 mutants carrying point mutations in their deaminase domain (e.g., mouse ADAR1 p150^E861A^), dsRBD (e.g., mouse ADAR1 p150^EAA^) or ZBD (e.g., mouse ADAR1 p150^P195A^) serve as ideal tools for distinguishing editing-dependent and editing-independent mechanisms. These mutants are referred to below as “editing-dead,” “binding-dead” or “zbd-dead,” respectively. Experimentally, combinatorial approaches—including re-introduction and co-knockout—can be employed to test functional sufficiency and expand signaling networks.

## 3 A-to-I editing and ADAR in normal hematopoiesis

### 3.1 Embryonic hematopoiesis

Current knowledge on embryonic hematopoiesis from ADAR1-related *in vivo* experiments is listed in Supplementary Table S1. ADAR1’s role was first indicated in 2000, when Wang et al. (2000) observed that the peripheral erythrocytes of live chimeric *Adar1* ^+/−^ embryos were arrested at mitosis phase, resulting in an absence of mature erythrocytes which might underlie hypoxia and the following embryonic lethality before embryonic day 14 (E14.0) (Wang et al., 2000). Subsequent studies reproduced the impaired hematopoiesis and embryonic lethality in *Adar1* ^+/−^ heterozygotes with gene manipulation (Hartner et al., 2004; Wang et al., 2004; Hartner et al., 2009; Pestal et al., 2015; Heraud-Farlow et al., 2017). Moreover, the equivalent phenotypes between *Adar1*-deficient and *Adar1 p150*-deficient embryos suggest a p150-dominant pattern (Ward et al., 2011; Pestal et al., 2015; Liang et al., 2023b). Actually, ADAR1 p150 is continuously required for embryonic and adult hematopoiesis (Liang et al., 2023b).

Embryonic hematopoiesis is sophisticated spatiotemporally. The initial wave, termed “primitive hematopoiesis,” occurs in yolk sac (YS) as early as E7.0 and gives rise to erythrocytes and macrophages. The adult-type hematopoiesis, termed “definitive hematopoiesis,” replaces primitive hematopoiesis around E8.25, in aorta-gonad-mesonephros region firstly and then in fetal liver (FL), thymus (Thy), spleen (Sp) and ultimately bone marrow (BM) (Golub and Cumano, 2013). Most studies suggest that the major role of ADAR1 is to facilitate definitive hematopoiesis, especially in FL, rather than primitive erythropoiesis in YS (Hartner et al., 2004; Wang et al., 2004; Mannion et al., 2014). *Adar1* deficiency affected neither the number of YS colony-forming unit-erythroid (CFU-E) progenitors at E8.5 (Hartner et al., 2004) nor the morphology of primitive erythrocytes in peripheral blood (PB) at E11.0 (Wang et al., 2004). In contrast, it enhanced apoptosis and reduced the number of definitive progenitors in various hematopoietic tissues at E10.5 (Wang et al., 2004) or E11.5 (Hartner et al., 2004; Liang et al., 2023b). *In vitro* cell culture and chimera studies reported that ADAR1 promoted the proliferation and survival of hematopoietic progenitors rather than their differentiation, in a cell-autonomous fashion (Hartner et al., 2004). However, FL hematopoietic cell transplantation revealed that *Adar1* deficiency impaired progenitor differentiation but did not affect HSC emergence in FL (Hartner et al., 2009). The contradiction may result from different embryonic stages or methodological differences between *in vitro* and *in vivo* experiments.

ADAR1-mediated RNA editing is instrumental to definitive erythropoiesis. At E12.5, *Adar1 p150* deficiency arrested FL erythropoiesis at R1 stage, attenuating both cell viability and maturation (Liang et al., 2023b). At E13.5, editing-dead *Adar1* caused erythropoiesis failure in FL, following decreased A-to-I editing frequency in erythroid lineage-specific transcripts and upregulated dsRNA response-related genes at E12.5 (Liddicoat et al., 2015). At E14.5, erythrocyte-specific *Adar1* depletion arrested FL erythropoiesis at R3 stage with upregulated ISGs (Liddicoat et al., 2016). In fact, Liddicoat et al. (2015) and Liddicoat et al., (2016) proposed that *Adar1* deficiency-related embryonic lethality might result from insufficient RNA editing which failed to attenuate immunogenicity of lineage-specific transcripts, such as the erythroid-specific *Klf1* transcript (Liddicoat et al., 2016).

When assessing ADAR1’s role in embryonic hematopoiesis, it is crucial to differentiate cell-intrinsic from microenvironmental effects. While cell-autonomous patterns are well-established (Hartner et al., 2004; Hartner et al., 2009; Liddicoat et al., 2016), *Adar1* deficiency also disrupts FL microenvironment—the key site for definitive hematopoiesis—through widespread hepatocyte apoptosis and structural defects (Hartner et al., 2004; Wang et al., 2004; Mannion et al., 2014). Therefore, ADAR1’s dual impacts on both hematopoietic cells and their supportive niche, coupled with its essential role in specific organogenesis (Hartner et al., 2004), contribute to embryonic lethality collectively.

Mechanistically, evidenced by concurrent gene manipulation, ADAR1’s function in embryonic hematopoiesis is solely dependent on RNA editing-mediated suppression of the MDA5-MAVS pathway (Mannion et al., 2014; Liddicoat et al., 2015; Pestal et al., 2015; Liddicoat et al., 2016; Heraud-Farlow et al., 2017), with no significant involvement of editing-independent functions (Liddicoat et al., 2016; Heraud-Farlow et al., 2017) or other antiviral sensors like PKR and RIG-I (Wang et al., 2004; Pestal et al., 2015). Actually, hematopoietic defects caused by *Adar1* deficiency may be largely attributed to MAVS activation and IFN production downstream of MDA5, given the insignificant transcriptomic differences between *Adar1* ^−/−^ *Mavs* ^−/−^ and *Adar1* ^+/+^ *Mavs* ^−/−^ embryonic livers (Mannion et al., 2014).

### 3.2 Adult hematopoiesis: hematopoietic stem and progenitor cells

The hematopoietic hierarchy consists of diverse cell types all derived from HSCs. During adult hematopoiesis, HSCs give rise to hematopoietic progenitor cells (HPCs) which have lost self-renewal capacity while retaining differentiation potential. The earliest HPCs are multi-potent progenitors (MPPs), and the multi-potency is restricted as MPPs generate oligo-potent progenitors including the common lymphoid progenitors (CLPs) and the common myeloid progenitors (CMPs). CLPs and CMPs subsequently differentiate into unipotent progenitors, also known as lineage-committed progenitors. The heterogeneous population enriched for stem cell activity is termed hematopoietic stem and progenitor cells (HSPCs), encompassing HSCs and early progenitors. In practice, mouse HSPCs are defined by a lineage marker (Lin)^−^c-Kit^+^Sca-1^+^ (LKS^+^) surface marker profile, while later differentiated populations display a Lin^+^c-Kit^+^Sca-1^−^ (LKS^−^) profile.

Several studies have demonstrated that ADAR1 is dispensable for HSC emergence, but crucial to HPC maintenance. As summarized in Supplementary Table S2, bone marrow transplantation experiments have established that *Adar1* deletion attenuated the hematopoietic reconstitution capacity by inducing apoptosis in differentiating HPCs (XuFeng et al., 2009). Mouse models reproduced the increased and decreased frequency of HSC-enriched LKS^+^ cells and HPC-enriched LKS^−^ cells in BM, respectively (Hartner et al., 2009; Nakahama et al., 2021). Moreover, *Adar1* deficiency specifically attenuated *in vitro* proliferation of LKS^−^ progenitors, but not LKS^+^ ones. Compatible with this selective role, ADAR1 expression was higher in HPCs than in HSCs or mature BM populations (XuFeng et al., 2009). Nevertheless, its effects on HSCs could not be completely excluded. Upon *in vitro Adar1* overexpression, both HSCs and HPCs exhibited increased proliferation, accelerated cell cycle transit and editing-dependent transcriptional alterations in cell cycle regulatory pathways (Jiang et al., 2019). Inducible *Adar1* deletion specifically decreased the number of LKS^+^CD34^low^ long-term HSCs (LT-HSCs), and increased the percentage of S-phase cells within LKS^+^CD34^high^ short-term HSCs (ST-HSCs) (Hartner et al., 2009). It is ambiguous whether such activation in the stem cell compartment reflects a compensatory response to pancytopenia or a direct consequence of ADAR1 loss. High-throughput sequencing provides novel insights into how RNA editome balances HSPC proliferation and differentiation. Both human (Wu et al., 2023) and mouse (Wang et al., 2021) RNA editomes are dynamic during adult hematopoiesis, with stable editing events (i.e., shared sites among HSPCs), group-specific editing events (i.e., classifying sites according to HSPCs’ differentiation potential) and stage-specific editing events (i.e., classifying sites according to cell type) (Wang et al., 2021) occurring in either HPC differentiation-related genes or cell cycle-related genes (Wang et al., 2021; Wu et al., 2023). RNA editing also influences cell fate decision, given that editing frequency at the lineage commitment point changed significantly (Wang et al., 2021), and lineage-specific editing sites were involved in specific differentiation pathways correspondingly (Wu et al., 2023).

Mechanistically, ADAR1’s editing function is indispensable for HPC survival, given the remained HPC defects upon re-introduction of editing-dead *Adar1* (XuFeng et al., 2009). Jiang et al. (2019) has elaborated on the role of editing-dependent regulation on miRNA in HSPC self-renewal: A-to-I editing in the DROSHA cleavage site impaired miR-26a biogenesis, thereby derepressing the expression of enhancer of zeste homolog 2 (EZH2), epigenetically downregulating cyclin dependent kinase inhibitor 1A (CDKN1A) and finally activating cell cycle transit (Jiang et al., 2019). Inspired by the MPP-specific recoding editing event in antizyme inhibitor 1 (Azin1) gene, Wang et al. (2021) elegantly revealed both *in vivo* and *in vitro* that recoded AZI protein, which was exclusively catalyzed by ADAR1 p150 (Xing et al., 2023), translocated to the nucleus where it bound to and altered the genomic distribution of DEAD boxpolypeptide1 (DDX1). The edited AZI-DDX1 complex was essential for murine HSPC differentiation by influencing the transcription of hematopoietic regulators (Wang et al., 2021). In human HSPCs, editing events in *Eif2ak2* (the gene encoding PKR protein) 3′UTR were associated with gene expression and differentiation potential. It may reflect a defensive preconditioning to rapidly initiate ISR and thus maintain cellular homeostasis during HSPC differentiation when cells are susceptible to stimuli (Wu et al., 2023). It is also hypothesized that IFN signaling hyperactivation, arising from insufficient RNA editing, would lead to persistent high expression of stem cell antigen-1 (Sca-1) which mediated IFN-induced HSC apoptosis (Hartner et al., 2009). Such a linker role of Sca-1 has been supported by the positive correlation between the frequency of apoptotic cells and Sca-1 expression level in *Adar1*-deficient mice (Hartner et al., 2009; Liang et al., 2023b). However, this unfavorable effect of IFN signaling is contradictory to its role in activating dormant HSCs (Essers et al., 2009). ADAR1’s non-editing functions also contribute to HSC homeostasis. For example, Z-RNA recognition promotes LKS^+^ to LKS^−^ progenitor differentiation, perhaps through maintaining ADAR1’s editing efficiency (Nakahama et al., 2021). Intriguingly, ADAR1 may cooperate with the apolipoprotein B mRNA-editing enzyme catalytic polypeptide-like 3 (APOBEC3), another deaminase catalyzing DNA C-to-T transitions, to regulate HSPC fate, as revealed by the skewed differentiation towards erythroid (Jiang et al., 2021) lineage or B-lymphoid lineage (Jiang et al., 2019; Jiang et al., 2021) upon *Apobec3* or *Adar1* overexpression, respectively.

Overall, A-to-I editing and ADAR are versatile in the complex hematopoietic niche, regulating and safeguarding the self-renewal, lineage commitment and differentiation of HSCs and HPCs. Identifying their key targets will possibly uncover crucial molecules for HSC homeostasis.

### 3.3 Adult hematopoiesis: T lymphopoiesis

T lymphopoiesis originates from CLPs in BM and proceeds within the thymic niche. Based on CD4 and CD8 surface expression, thymocytes are classified as double negative (DN), double positive (DP), or single positive (SP) populations. The DN population is subdivided into four stages termed as DN1, DN2, DN3 and DN4, with T cell receptor (TCR) β (and TCRγδ) rearrangement occurring at DN3 stage. Successfully rearranged DN3 cells carry a pre-TCR complex while failed ones undergo apoptosis. TCRα arrangement takes place at DP stage and produces a TCR complex for positive selection and negative selection, aiming to select DP cells interacting with major histocompatibility complex (MHC) and tolerating self-peptide, respectively. Qualified DP cells differentiate into CD8^+^ SP or CD4^+^ SP, finally emigrating to the periphery (Kumar et al., 2018).

ADAR1 is essential to thymocyte development and maturation (Supplementary Table S3). *Cd4* ^+^ T cell-specific *Adar1* deletion disrupted DP-to-SP transition and triggered autoimmunity, manifested as spontaneous colitis with localized T cell accumulation in mice (Nakahama et al., 2018). In contrast, *Lck* ^+^ (lymphocyte cell-specific protein-tyrosine kinase) T cell-specific *Adar1* deletion arrested earlier lymphopoiesis, likely at DN3-to-DN4 transition (Vongpipatana et al., 2020; Xufeng et al., 2020). The precise developmental blockade remains unclear due to incomplete deletion efficiency (Xufeng et al., 2020).

Consensus holds that MDA5-MAVS signaling mediates ADAR1’s role in T cell development. ADAR1’s inhibition on downstream ISG expression ensures proper TCR signal transduction (Nakahama et al., 2018; Vongpipatana et al., 2020; Xufeng et al., 2020), which is important to maintain cell survival (Vongpipatana et al., 2020), orchestrate differentiation (Nakahama et al., 2018; Vongpipatana et al., 2020; Xufeng et al., 2020) and establish thymic self-tolerance (Nakahama et al., 2018; Vongpipatana et al., 2020). Accordingly, concurrent deletion of *Adar1* and *Ifih1* (Nakahama et al., 2018; Vongpipatana et al., 2020) or *Mavs* (Pestal et al., 2015) at least partially restored thymocyte development. Intriguingly, such suppression contributes to thymic selection. Nakahama et al. (2018) elegantly demonstrated that *Adar1* deficiency impaired both negative and positive selection, as the number of CD8^+^ SP thymocytes in male and female *Cd4* ^Cre^ *Adar1* ^fl/fl^ HY-TCR^+^ mice was higher and lower compared with that in HY-TCR^+^ mice, respectively. Further analysis revealed that the attenuated TCRβ signaling, possibly caused by ISG overexpression in *Adar1*-deficient thymocytes, mediated compromised T cell selection, aberrant thymocyte maturation and dysregulated autoimmune response (Nakahama et al., 2018). Besides the “trainee,” ADAR1 also affects the “trainer” in negative selection by diversifying the self-antigen repertoire, given that RNA editing level in medullary thymic epithelial cells (mTECs) was relatively high (Danan-Gotthold et al., 2016), and *Adar1* deletion reduced mTECs (Vongpipatana et al., 2020).

MDA5-dependent and -independent mechanisms may coordinate non-redundantly to regulate T lymphopoiesis. For example, activated MDA5 signaling caused by *Adar1* deletion triggered ISG expression and cell apoptosis, whereas MDA5-independent impairment of TCRβ signaling preferentially disrupted DN3-to-DN4 transition. Supporting this complementarity, forced TCR expression together with *Ifih1* deletion efficiently rescued developmental defects in *Adar1* ^−/−^ mice, whereas neither intervention alone was sufficient (Vongpipatana et al., 2020). However, the salvage was far from equivalence with controls, indicating other mechanisms regulating T cell development.

Regarding A-to-I editing, murine editome analysis revealed that during DP-to-4SP transition, A-to-I editing frequency in wild type thymocytes increased significantly, which was consistent with elevated expression of total ADAR1, ADAR1 p150, MDA5 and ISGs (Nakahama et al., 2018). Nevertheless, editing-dead mouse model has excluded the requirement of RNA editing for MDA5-unrelated pathways (Nakahama et al., 2018; Vongpipatana et al., 2020).

### 3.4 Adult hematopoiesis: B lymphopoiesis

B lymphopoiesis proceeds through sequential stages in BM, including pro-B stage, large pre-B stage, small pre-B stage, and immature B stage, before migration to peripheral lymphoid organs for final maturation. The pre-B-cell receptor (pre-BCR) and B-cell receptor (BCR), serve as critical checkpoints expressed on large pre-B cells and immature B cells, respectively.

ADAR1 is required for both central B lymphopoiesis and peripheral maturation (Supplementary Table S3). Centrally, B cell-specific *Adar1* deletion not only arrested B lymphopoiesis before immature stage *in vivo*, but also attenuated *in vitro* differentiation potential of pro-B cells (Marcu-Malina et al., 2016; Chen et al., 2022). And *Adar1* overexpression in HSPCs increased B cell expansion (Jiang et al., 2019; Jiang et al., 2021). Peripherally, B lymphopenia was observed in PB (Marcu-Malina et al., 2016; Chen et al., 2022; Liang et al., 2023b) and Sp (Pestal et al., 2015; Liang et al., 2023b) of *Adar1* ^−/−^ mice. It is likely that ADAR1 regulates the differentiation of early precursors and meanwhile, promotes the survival of late B populations. Notably, ADAR1 p150 is required for germinal center B cell response, T cell-dependent antibody secretion and memory B cell formation upon immunization (Li et al., 2022). To date, this remains the only study examining ADAR1’s function under immune challenge, underscoring its continuous requirement in maintaining homeostasis.

Similar to its role in T lymphopoiesis, ADAR1 regulates B lymphopoiesis through both MDA5-dependent and -independent mechanisms, which jointly support cell survival and differentiation. An isoform-specific, editing-independent pattern is striking. ADAR1 p150, the predominant isoform expressed in B cell subsets (Chen et al., 2022; Li et al., 2022), promoted early B cell survival from early pro-B to large pre-B cell stage through MDA5 suppression. It also enhanced IgH transcription and pre-BCR expression during the large-to-small pre-B transition through MDA5-independent mechanism, which did not involve PKR or RNase L pathways (Chen et al., 2022). Additionally, MDA5-independent pathways are indispensable for peripheral maturation given the incomplete restoration of mature B cell number (Pestal et al., 2015) and B cell response (Li et al., 2022) after concurrent *Ifih1* deletion. Importantly, the binding activity of ADAR1 p150 is essential throughout B lymphopoiesis, irrespective of downstream pathways (Chen et al., 2022; Li et al., 2022; Liang et al., 2023b). In early development, the binding process was mediated by the canonical dsRBD, suggesting potential redundancy with other RNA-binding proteins such as ADAR1 p110 (Chen et al., 2022). However, in peripheral maturation, ADAR1 p150 appeared uniquely capable of activating B cell responses, as neither nuclear nor cytoplasmic ADAR1 p110 could do so in *Adar1* ^−/−^ mice (Li et al., 2022), implying isoform-specific functional distinction beyond mere dsRNA binding capacity.

## 4 A-to-I editing and ADAR in leukemia

**FIGURE 4:**
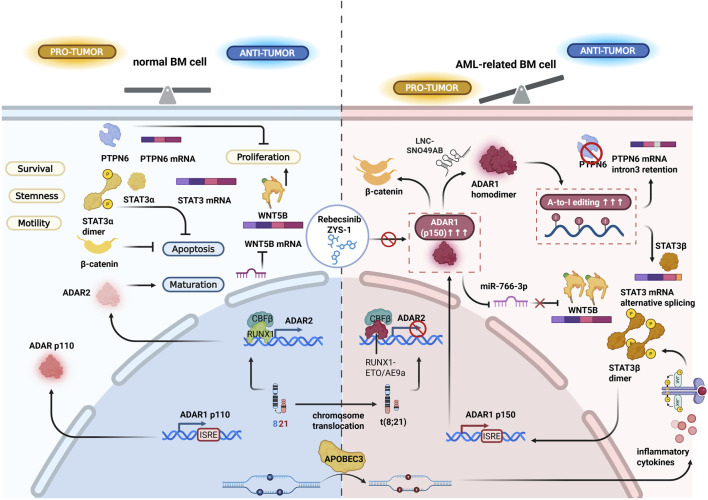
The role of A-to-I editing and ADAR1 in AML. Cytogenetic abnormalities, along with inflammatory microenvironment, upregulate and downregulate ADAR1 (p150) and ADAR2 in AML-related cells, respectively, thereby disrupting the balance between pro-tumor and anti-tumor effects. Increased ADAR1 (p150) and A-to-I editing promotes leukemogenesis and disease progression by altering splicing and influencing miRNA activity, thereby dysregulating proliferation and apoptosis pathways. ADAR1 p150 has been proposed as a therapeutic target, with its inhibitors showing anti-leukemic activity in pre-clinical studies. Created with www.biorender.com.

**FIGURE 5:**
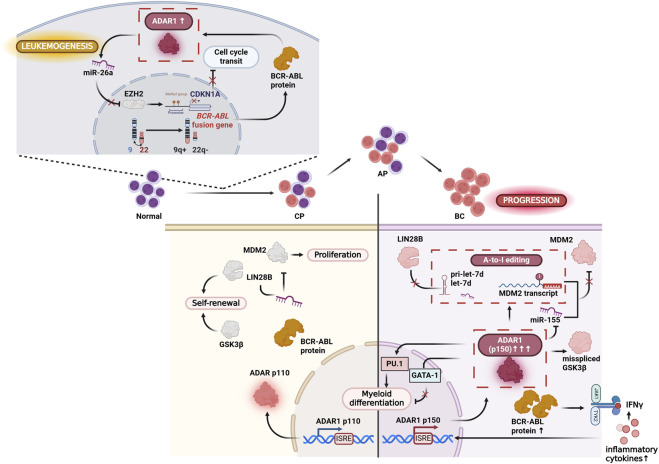
The role of and A-to-I editing and ADAR1 in CML leukemogenesis and progression. Dysregulated ADAR1 in BCR-ABL^+^ cells accelerates cell cycle transit by antagonizing tumor-suppressive miRNA activity. During CP-to-BC transformation, BCR-ABL-driven upregulation of ADAR1 (p150) enhances leukemia stem cell self-renewal, promotes proliferation and skews differentiation by altering splicing, impairing miRNA activity and dysregulating transcription factors. Notably, both editing mechanism and non-editing mechanism contribute to CML progression. Created with www.biorender.com.

**FIGURE 6:**
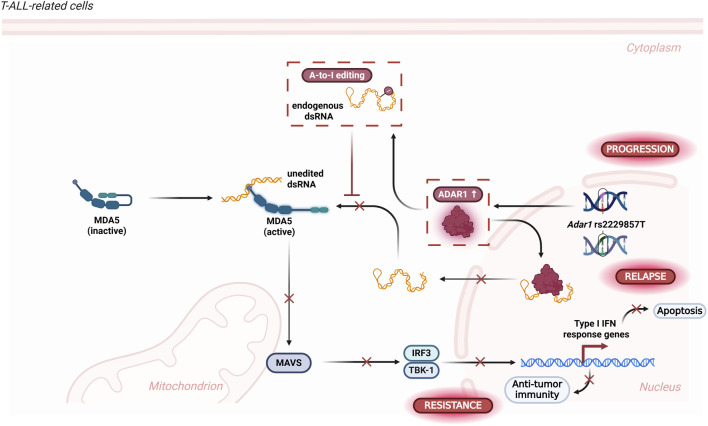
The role of A-to-I editing and ADAR1 in ALL. In T-ALL, upregulated ADAR1, possibly resulting from single-nucleotide polymorphism of *Adar1* gene, inhibits tumor-suppressive protein via miRNA editing. It also contributes to treatment resistance and leukemia relapse through suppressing MDA5-mediated dsRNA sensing, thereby reshaping a tumor-suppressive microenvironment. Created with www.biorender.com.

**FIGURE 7:**
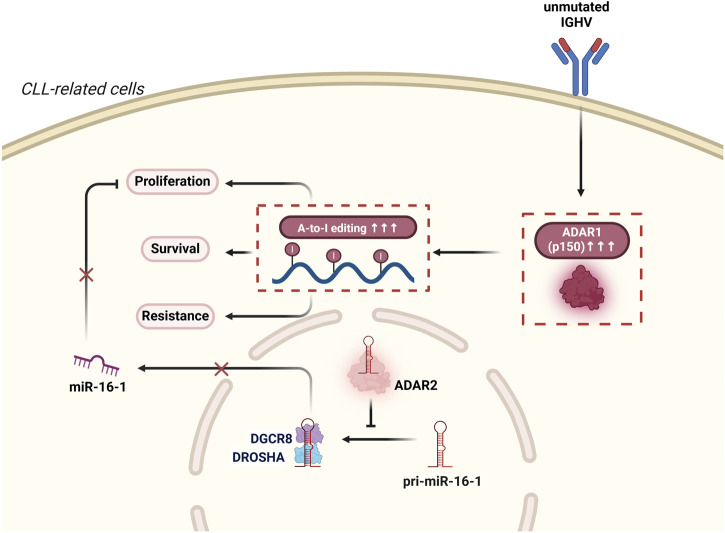
The role of A-to-I editing and ADAR1 in CLL. In IGHV-unmutated CLL cells, increased ADAR1 (p150) and A-to-I editing promotes cell survival, proliferation and resistance with unknown mechanism. ADAR2 also plays a pro-tumor role through editing-independent impairment of tumor-suppressive miRNA processing. Created with www.biorender.com.

Leukemia is a clonal hematologic malignancy arising from HSCs or HPCs, characterized by uncontrolled proliferation and impaired differentiation of abnormal hematopoietic cells in BM, PB and lymphoid tissues. Leukemia stem/initiating cells (LSCs/LICs) are a subpopulation of leukemia cells functionally defined by the ability to propagate leukemia after xenotransplantation in immunodeficient mice. Driven by cytogenetic abnormalities, the self-renewal capacity of these malignant stem cells underpins disease initiation, maintenance, relapse and therapeutic resistance. Given the established roles of A-to-I editing and ADAR in normal hematopoiesis, it is reasonable to speculate that aberrant A-to-I editing and ADARs contribute to leukemic hematopoiesis. As summarized in Table 1, clinical observational studies have consistently reported alteration of A-to-I editing level and ADAR expression level in patient leukemia cells, some of which correlate with disease progression and prognosis. Intriguingly, leukemia cells exhibit remarkable heterogeneity in editing patterns, which may be linked to underlying cytogenetic features. As illustrated in Figures 4–7, wet experiments partially addressed the molecular pathogenesis of the alteration and further revealed its effects on leukemia cell survival and proliferation with various mechanisms, hinting translational potential of ADAR-targeted therapy. However, unlike substantial evidence in normal hematopoiesis, to date, only one study in T-cell acute lymphoblastic leukemia has validated the ADAR1-dsRNA response axis in leukemia. In this section, we will focus on findings from animal/cell line experiments, delineating the existence, pathogenesis, functional consequences and therapeutic potential of aberrant A-to-I editing and ADARs in each leukemia subtype.

**TABLE 1:** Clinical observational studies on A-to-I editing and ADARs in leukemia.

Disease	Editing/Enzyme alteration	Clinical relevance	Editome heterogeneity
AML	A-to-I editing↑(Meduri et al., 2022) ADAR1↑ (Xiao et al., 2019; Shi et al., 2025) ADAR2↓(Meduri et al., 2022)	- Higher leukemia cell proportion associated with: higher ADAR1 (Xiao et al., 2019) - Poorer prognosis associated with: ① higher A-to-I editing (Meduri et al., 2022); ②higher ADAR1 (Shi et al., 2025); ③ SOCS2-AS1 editing (Meduri et al., 2022)	- High-risk genotypes vs. Low-risk ones: A-to-I editing↑(Meduri et al., 2022) - AML vs. AML CR: ADAR1↑ (Rossetti et al., 2017) - CBF AML vs. Non-CBF AML: ① ADAR2↓(Guo et al., 2023); ② ADAR2-targeted A-to-I editing↓ (Guo et al., 2023)
sAML	A-to-I editing↑(Jiang et al., 2021) 3′UTR and intronic editing↑ (Jiang et al., 2021) ADAR1 p150/p110 ratio↑ (Jiang et al., 2021)	​	​
CML	ADAR1↑(Steinman et al., 2013) ADAR1 p150↑(Jiang et al., 2013)	​	- BC vs. CP: ① A-to-I editing↑ at certain sites (Jiang et al., 2013; Crews et al., 2015; Zipeto et al., 2016); ② 3′UTR A-to-I editing↑(Jiang et al., 2019); ③ ADAR1↑(Jiang et al., 2019), ADAR1 p150↑(Jiang et al., 2013)
B-ALL	ADAR1 p110↑(Ma et al., 2011)	- Higher incidence associated with: ADAR1 SNP (Ma et al., 2011) - Poorer prognosis associated with: lower ADAR1 expression (Ma et al., 2011) - Higher relapse risk associated with: ADAR1 SNP (Ma et al., 2011)	- CR vs. ND: ADAR1 p110↓ (Ma et al., 2011)
T-ALL	ADAR p150↓(Ma et al., 2011) Different miRNA editome (Xie et al., 2022) ADAR1 p150↑(Rivera et al., 2024)	- Higher relapse risk associated with: higher editing level (Rivera et al., 2024) - Higher leukemia-associated mortality associated with: higher editing level (Rivera et al., 2024)	- Relapsed vs. Non-relapsed: A-to-I editing↑(Rivera et al., 2024) - Immature cells vs. Differentiated ones: ADAR1↑(Rivera et al., 2024)
CLL	ADAR1 p150↑(Gassner et al., 2021) More varied editing levels (Gassner et al., 2021) Different RNA editome (Gassner et al., 2021)	- Poorer prognosis of IGHV-mutated patients associated with: lower A-to-I editing level (Gassner et al., 2021) - Shorter PFS associated with: miR-3157 editing (Gassner et al., 2021)	- IGHV-unmutated vs. IGHV-mutated: ① different RNA editome (Gassner et al., 2021); ② A-to-I editing ↓(Gassner et al., 2021); ③ ADAR↑, ADAR p110↑, ADAR p150↑(Gassner et al., 2021)

“Editing/Enzyme alteration” denotes differences between leukemia patients and healthy controls. “Editome heterogeneity” denotes differences among leukemia patients. Abbreviations: AML, acute myeloid leukemia; sAML: secondary AML; CML: chronic myeloid leukemia; B-ALL: B-cell acute lymphoblastic leukemia; T-ALL: T-cell acute lymphoblastic leukemia; CLL: chronic lymphoblastic leukemia; CR: complete remission; ND: newly diagnosed; BC: blast crisis; CP: chronic phase; CBF: core-binding factor; CBF AML: AML, patients carrying abnormal CBF: ; IGHV: immunoglobulin heavy chain variable region; PFS: progression-free survival.

### 4.1 Acute myeloid leukemia (AML)

Research on RNA editing in AML dates back to 2000. The mechanisms driving aberrant RNA editing in AML are diverse. The t (8;21) translocation, resulting in runt-related transcription factor 1 (RUNX1) fusion protein, represents cytogenetics-driven mechanism: acting in a dominant-negative manner, RUNX1 fusion protein competed with normal RUNX1 protein for binding *Adar2* DNA, thereby downregulating ADAR2 and ADAR2-mediated editing. This also explains altered ADAR2 activities in individuals carrying abnormal core binding factor (CBF) which is a key transcription regulator in hematopoeisis composed of RUNX1 and CBFβ (Guo et al., 2023). In contrast, the lncRNA LNC-SNO49AB promoted ADAR1 homodimerization by binding to its dsRBDs, thereby facilitating A-to-I editing in AML cells (Huang et al., 2022). Additionally, quantitative trait locus analyses suggested that transcription factors and RNA-binding proteins might also regulate RNA editing in AML (Meduri et al., 2022).

ADAR1’s and ADAR2’s editing functions play opposite roles in AML pathogenesis (Figure 4), possibly stemming from their unique RNA substrates and independent activities (Rossetti et al., 2017). ADAR1-mediated RNA editing contributes to AML pathogenesis by altering pathways governing proliferation, differentiation, and apoptosis. Aberrant A-to-I editing can recode key proteins involved in proliferation and apoptosis (Quelen et al., 2016; Meduri et al., 2022). It can also cause intron retention in protein tyrosine phosphatase non-receptor type 6 (Ptpn6) transcripts, thereby attenuating PTPN6’s negative regulation of proliferation and finally promoting leukemogenesis and invasiveness (Beghini et al., 2000). ADAR1-mediated editing also accelerates secondary AML (sAML) pathogenesis by integrating environmental stimuli and genomic instability, specifically from pre-leukemia stem cells (pre-LSCs) to LSCs: against a background of APOBEC3-mediated C-to-T DNA mutations, inflammation-responsive ADAR1 p150 in pre-LSCs drove LSC evolution with extensive editome changes, such as redistributed editing events towards 3′UTR, and distinctive editing events in ribosomal regulatory transcripts. Notably, intronic editing of signal transducer and activator of transcription 3 (Stat3) transcripts induced alternative splicing, and the resultant STAT3β isoform facilitated STAT3 phosphorylation. Phosphorylated STAT3 acted as a self-renewal agonist, promoting LSC evolution and expansion. Given that JAK/STAT signaling upregulated ADAR1, there might be a positive feedback loop accelerating pre-LSC to LSC transformation, underscoring the therapeutic potential of targeting JAK/STAT pathway in sAML (Jiang et al., 2021). In contrast, ADAR2-mediated RNA editing may prevent AML pathogenesis—at least in those carrying RUNX1 fusion—by recoding proteins rather than regulating gene transcription. Specifically, two known recoded proteins involved in protein transport—component of oligomeric golgi complex 3 (COG3) and coat protein complex i subunit alpha (COPA)—exhibited anti-leukemic capability following re-introduction of either wild-type ADAR2 or edited transcripts (Guo et al., 2023).

Consistent with above editing-associated distinctions, ADAR1 and ADAR2 themselves also play complementary roles in AML. ADAR1 is essential for cell stemness, as evidenced by its ubiquitous expression in patient blasts (Rossetti et al., 2017; Xiao et al., 2019; Shi et al., 2025), the positive correlation between its mRNA level and leukemia cell proportion (Rossetti et al., 2017; Xiao et al., 2019), and the extensive cell death following its silencing (Rossetti et al., 2017; Shi et al., 2025). ADAR1 also contributes to AML cell proliferation by promoting Wnt signaling pathway (Xiao et al., 2019; Shi et al., 2025), mediated by editing-independent miRNA impairment and the following translational derepression (Shi et al., 2025). In contrast, ADAR2 is dispensable for AML cell proliferation and differentiation, but may promote post-differentiation maturation (Rossetti et al., 2017). An *in vitro* study using U937 cells highlights these differences: while ADAR1 mRNA and protein levels increased during differentiation, ADAR2 mRNA level was lower than ADAR1’s and declined throughout differentiation. Surprisingly, ADAR2 protein level surged post-differentiation, whereas ADAR1 level increased progressively. This transcription-translation discrepancy for ADAR2 might be attributed to ubiquitin-proteasome-mediated protein degradation (Rossetti et al., 2017), supporting that ADAR2 is unnecessary and even potentially detrimental to AML blast proliferation and differentiation.

How RNA editing contributes to RNA homeostasis and interacts with broader transcriptional dysregulation in AML remains a crucial topic for investigation. While it has minimal impact on global transcription level (Meduri et al., 2022; Guo et al., 2023), site-specific analyses reveal a complex picture: most recurrent A-to-I editing sites in AML patients showed an inverse correlation between editing frequency and transcript abundance, yet gene expression was higher in patients with specific editing events compared to those lacking them (Meduri et al., 2022). Meduri et al. (2022) propose that this paradox reflects RNA editing’s role as a cis-acting regulator within a feedback loop to maintain transcription homeostasis, where transcript accumulation triggers editing to suppress transcription itself. This hypothesis currently lacks experimental support.

Collectively, above distinctions of ADAR1 and ADAR2 suggest pro-tumorigenic and tumor-suppressive roles, respectively. Encouragingly, ADAR-targeted strategies have shown translational potential. ZYS-1, an adenosine analog presented as an ADAR1 inhibitor, significantly suppressed AML cell proliferation both *in vivo* and *in vitro* (Shi et al., 2025). Nevertheless, one recent study refuted the role of ZYS-1 in ADAR1 inhibition (Smoak et al., 2025), thus questioning the efficacy of non-seletive ADAR1 inhibition. Inspired by the inflammation-responsive ADAR1 p150 overexpression in AML patients, Crews et al. (2023) developed a selective ADAR1 p150 small molecule inhibitor, Rencenib, and demonstrated its efficacy and safety: Rencenib reversed the *Adar1* isoform switch, reduced A-to-I editing, impaired self-renewal and anti-apoptotic capacity of LSCs, and consequently prolonged the survival of human AML xenograft mice while sparing normal hematopoiesis (Crews et al., 2023). Regarding ADAR2, *in vitro Adar2* overexpression inhibited CBF-AML cell (i.e., cells carrying abnormality in core binding factor) growth through recoding editing. In a t (8; 21) AML mouse model, wild-type *Adar2*, but not an editing-dead mutant, exerted anti-leukemic effects, including reduced undifferentiated BM cells, improved PB parameters, and prolonged survival (Guo et al., 2023). However, these tumor-suppressive effects of ADAR2 were not observed in non-CBF AML cells (Guo et al., 2023), indicating that the efficacy of ADAR2 targeting is cytogenetically context-dependent.

### 4.2 Chronic myeloid leukemia (CML)

CML progresses with three stages: chronic phase (CP), accelerated phase (AP) and blast crisis (BC). Early identification and timely prevention of CP-to-BC progression is critical for improving survival. Extensive studies have explored the significant differences in A-to-I editing between CP and BC, with specific editing events contributing to CML progression (discussed below). Regarding the pathogenesis of the alteration, one prevailing hypothesis is that ADAR1 may be employed to amplify leukemogenic effects of the Philadelphia chromosome t (9;22) (q34;q11) (Ph), the cytogenetic abnormality carried by approximately 95% CML patients. During CP-to-BC transition, the Ph-caused BCR-ABL amplification promoted ADAR1 expression through activating IFN-related pathways (Jiang et al., 2013; Steinman et al., 2013; Zipeto et al., 2016). Functional evidence further supported it: CML cell-specific *Adar1* knockout decreased CD34^+^CD38^+^Lin^−^ progenitor cells harboring BCR-ABL fusion, and reversed CML phenotypes (Jiang et al., 2013).

As a cytogenetic “amplifier,” ADAR1 regulates CML stem/progenitor cell self-renewal and influences CML progression, by altering transcription factor (TF) activation, causing alternative splicing and disrupting miRNA targeting. Notably, the editing and non-editing activities orchestrate the CP-to-BC transition and will therefore be discussed integrally. *Adar1* knockout in immunodeficient mice remarkably decreased BM engraftment of secondary transplantation while neither leukemia burden nor primary transplantation was affected, indicating impaired self-renewal (Jiang et al., 2013). ADAR1 also upregulated myeloid TF PU.1, downregulated erythroid TF GATA-1, and increased a misspliced form of glycogen synthase kinase-3 β (GSK3β), thereby reprogramming myeloid progenitor differentiation and accelerating CML progression (Jiang et al., 2013). Two miRNA-associated axes contribute to CP-to-BC transition: the LIN28B/let7 axis and the MDM2/miR-155 axis. ADAR1 disrupted let-7d miRNA biosynthesis through editing-dependent mechanism, alleviating miRNA repression of oncogenic proteins, including Lin28 Homology B (LIN28B), which ultimately enhanced self-renewal capacity and accelerated BC progression. In particular, a BC-specific +3 A-to-I editing in pri-let-7d miRNA was predicted to alter pri-miRNA secondary structure, thereby impairing miRNA maturation (Zipeto et al., 2016). Another downregulated miR-26a, following *in vitro* ADAR1 activation, also increased LIN28B expression in BC progenitors (Jiang et al., 2019), suggesting parallel oncogenic pathways regulated by ADAR1. Concerning the other axis, ADAR1 altered miRNA targeting sites by 3′UTR editing in *Mdm2* transcripts and impaired miR-155 biogenesis through an editing-independent mechanism, thereby facilitating MDM2 expression and its pro-tumor role, as evidenced by irreversible cell proliferation upon co-transfection of miR-155 and edited *Mdm2* (Jiang et al., 2019). Furthermore, low expression of miRNA processing-related genes was reported in BC patient progenitor cells (Zipeto et al., 2016), consistent with above ADAR1-mediated impaired miRNA biogenesis. Beyond CML progression, ADAR1’s editing activity also contributes to leukemogenesis through impairing miR-26a maturation, which derepressed EZH2 expression and indirectly downregulated CDKN1A, thereby accelerating cell cycle transit of normal hematopoietic progenitors (Jiang et al., 2019). As discussed earlier, the impaired miR-26a also benefited the self-renewal of normal HSPCs, and further bioinformatic analysis revealed that miR-26a targets were different between normal HSPCs overexpressing ADAR1 and CML CP progenitors, suggesting divergent effects of miR-26a in normal versus malignant progenitors (Jiang et al., 2019).

Taken together, the close ADAR1-CML interaction (Figure 5) lays a theoretical foundation for ADAR1-targeted therapy. Pre-clinical studies have shown promising efficacy and safety: *Adar1* knockout suppressed CML stem cell self-renewal (Jiang et al., 2013; Zipeto et al., 2016) and reversed murine CML phenotypes (Steinman et al., 2013; Zipeto et al., 2016), whereas it spared normal BM progenitors (Zipeto et al., 2016). Notably, the inhibitory effect might depend on ADAR1 expression level given that ADAR1 inhibitors did not change the number of LKS^+^ cells without BCR-ABL fusion or without *Adar1* overexpression (Steinman et al., 2013).

### 4.3 Acute lymphoblastic leukemia (ALL)

ALL is the most common cancer in children. To date, only two studies have directly explored RNA editing in ALL while others focus on ADAR1. The incidence of A-to-I editing is positively associated with the relapse risk in T-cell ALL (T-ALL), and ADAR1 isoform abundance is strikingly disease-specific and disease stage-specific in ALL patients (Table 1). The SNPs within *Adar1* gene may drive these alterations, as Cai et al. (2023) reported that Chinese children carrying rs9616T or rs2229857T alleles showed increased ADAR1 expression in PB and higher pALL incidence. Moreover, *Adar1* rs2229857T caused missense mutation and was significantly correlated with ALL relapse (Cai et al., 2023).

Despite the scarcity of functional studies, the few available ones offer key insights into how A-to-I editing and ADARs contribute to ALL, with a particular focus on the dsRNA response. Rivera et al. (2024) conducted in-depth investigation on ADAR1’s roles in T-ALL LICs with *in vivo* patient-derived xenograft (PDX) model, *in vitro* cell assays and the artificial thymic organoid system. They demonstrated that ADAR1 prevented apoptosis and promoted self-renewal of LICs by attenuating the dsRNA response with both editing-dependent and binding-dependent mechanisms. Specifically, the hyper-editing of cytosolic dsRNAs interfered with dsRNA detection by MDA5, and the binding of nuclear dsRNAs sequestered them from cytosolic MDA5. In contrast, binding-dependent mechanism might be sufficient to suppress another dsRNA sensor, PKR, which was briefly mentioned in the study. Beyond ADAR1’s pro-oncogenic roles in LICs, diverse rescue effects were observed when performing concurrent knockdown of ADAR1 and MDA5 in different PDX models and in different T-ALL cell lines. Furthermore, the intrinsic MDA5 levels paralleled different levels of the dependence on the ADAR1-dsRNA-MDA5 axis, while the PKR levels were comparable. Collectively, these findings suggest that ADAR1 inhibition may become a novel strategy to prevent relapse and overcome resistance in T-ALL, with intrinsic MDA5 level as a potential biomarker for therapeutic efficacy. Additionally, miRNA may also mediate the role of RNA editing, given the significantly different miRNA editing profiles between T-ALL cells and normal thymocytes (Xie et al., 2022).

### 4.4 Chronic lymphoblastic leukemia (CLL)

The immunoglobulin heavy chain variable region (IGHV) mutation status serves as an important prognostic factor for CLL. IGHV-unmutated cases usually indicate rapider disease progression and poorer outcomes. Clinical studies reveal tight correlation between A-to-I editing/ADAR1 and IGHV mutation in CLL (Table 1). However, the origin of such striking heterogeneity remains unclear, as no study has yet clarified the causal relationships underlying the association.

The role of A-to-I editing and ADAR may be mediated by miRNA in CLL progression. Although miRNA level was not associated with RNA editing, some miRNA editing events could act as survival predictors independent of IGHV mutation (Gassner et al., 2020). Interestingly, upregulated ADAR2 interfered with tumor-suppressive miRNA (i.e., pre-miR-16-1) processing in MEC-2 CLL cells through an editing-independent mechanism in which ADAR2 possibly competed against DROSHA by its dsRBD (Figure 7) (Allegra et al., 2014).

Regarding CLL treatment, RNA editing is characterized as a positive regulator for drug resistance. MEC1 cells with *Adar* knockout or RNA editing inhibition exhibited lower proliferation, decreased cell viability and improved sensitivity to CLL medications (Gassner et al., 2021). In addition, Xie et al. (2022) observed that radiotherapy reduced 3′-terminal adenosine editing levels of hsa-mir-21-5p and hsa-mir-155-5p in CLL cells.

## 5 The ADAR1-dsRNA response axis in hematology

The ADAR1-dsRNA response axis is strikingly context-dependent in hematology. During normal hematopoiesis, the axis is generally required to maintain homeostasis and ensure hematopoietic continuum. In contrast, although still unexplored, the requirement of the axis may exhibit intra-leukemia and inter-leukemia heterogeneity, and via promoting LIC stemness, the axis exerts pro-tumor effects on axis-dependent malignant cells. As summarized in Table 2, the ADAR1 p150-MDA5-IFN axis has been most extensively validated, whereas the ADAR1-PKR-ISR axis and other MDA5-independent signaling may synergize in different contexts. The dependence on RNA editing activity also varies.

**TABLE 2:** Summary on key phenotypes and mechanistic findings in normal hematopoiesis and leukemia.

Phenotype	ADAR protein (I)	Editing-dependent (II)	Editing-independent (II)	dsRNA sensor (III)	Strongest evidence
Embryogenesis/embryonic definitive hematopoiesis in fetal liver	ADAR1 p150	Yes	No	MDA5 PKR (excluded) RIG-I (excluded)	I: Isoform-specific KO Liang et al. (2023b) II: Editing dead KI Liddicoat et al. (2015), Liddicoat et al. (2016) III: Concurrent KO Liddicoat et al. (2015)
Adult HPC survival	ADAR1	Yes	​	​	I, II: Editing-dead mutant transduction XuFeng et al. (2009)
Adult HSPC self-renewal	ADAR1	Yes	​	​	I, II: Editing-dead KI Jiang et al. (2019)
Adult HSPC differentiation	ADAR1 p150	Yes	​	​	I, II: Edited target KI Wang et al. (2021)
Early T lymphopoiesis	ADAR1	​	​	MDA5	I, III: Concurrent KO Nakahama et al. (2018)
Thymic selection and self-tolerance	ADAR1	​	​	MDA5	I, III: Concurrent KO Nakahama et al. (2018)
Central B lymphopoiesis	ADAR1 p150 ADAR1 p110 (excluded)	No	Yes, dsRBD	MDA5 PKR (excluded) RNaseL (excluded)	I: Isoform-specific KI Chen et al. (2022) II: Editing-dead/binding-dead KI Chen et al. (2022) III: Concurrent KO Chen et al. (2022)
Peripheral B maturation	ADAR1 p150	​	​	MDA5 PKR (excluded) RNaseL (excluded)	I: Isoform-specific KO Pestal et al. (2015), Liang et al. (2023b) I: Isoform-specific KI Chen et al. (2022) II: Editing-dead/binding-dead KI Chen et al. (2022) III: Concurrent KO Chen et al. (2022)
Germinal center B cell response T cell-dependent antibody response	ADAR1 p150 ADAR1 p110 (excluded)	No	Yes, dsRBD	MDA5 PKR (excluded) RNaseL (excluded)	I: Isoform-specific KI Li et al. (2022) II: Editing-dead/binding-dead KI Li et al. (2022) III: Concurrent KO Li et al. (2022)
sAML: alternative splicing of Stat3 in LSC	ADAR1 p150	Yes	​	​	Editome analysis Jiang et al. (2021) II: RES detection Jiang et al. (2021)
AML: Wnt signaling upregulation	ADAR1	No	Yes	​	I, II: Editing-dead/binding-dead mutant transduction Shi et al. (2025)
CBF^+^AML: decreased recoded COG3 and COPA	ADAR2	Yes	​	​	I, II: Editing-dead/binding-dead mutant transduction Guo et al. (2023) I, II: Edited target KI Guo et al. (2023)
CML: let-7d miRNA biosynthesis	ADAR1	Yes	No	​	I, II: Editing-dead mutant transduction Zipeto et al. (2016)
CML: miR-155 biosynthesis	ADAR1	Yes	Yes	​	I, II: Editing-dead mutant transduction Jiang et al. (2019)
CML: MDM2 upregulation	ADAR1	Yes	Yes	​	I, II: Editing-dead mutant transduction Jiang et al. (2019) I, II: Edited target transduction Jiang et al. (2019)
ALL: LIC stemness	ADAR1 p150	Yes	Yes	MDA5 PKR	I, II: Editing-dead mutant transduction Jiang et al. (2019) I, III: Concurrent KO Rivera et al. (2024)
CLL: miR 16-1 biosynthesis	ADAR2	No	Yes, dsRBD	​	I, II: Editing-dead/binding-dead mutant transduction Allegra et al. (2014)

For normal hematopoiesis, phenotypes are described at the cellular level; for leukemia, at the molecular level. The strongest experimental evidence supporting the requirement of ADAR, proteins, the dependence on editing activity, and the involvement of dsRNA sensors is marked with I, II, and III, respectively.

We attempt to explain above context-dependence from three layers. First, why does the requirement of the axis seem shifted from normal hematopoiesis to malignant status. Fine-tuned differentiation and stemness maintenance are two goals of normal hematopoiesis, which simultaneously requires drivers and shields to regulate intrinsic programming and resist extrinsic challenges such as viral infection. Consequently, the ADAR1-dsRNA response axis fulfills these requirements since its role in self/non-self discrimination not only ensures cellular homeostasis for intricate biological processes, but also protects against high-risk false activation due to preventive upregulation of elements in dsRNA response (Wu et al., 2018). However, characterized by uncontrolled proliferation and impaired differentiation, leukemia represents an extremely dysregulated status with intra- and inter-individual cytogenetic heterogeneity. Reasonably, the malignancy might employ other pathways dependent on its specific cytogenetic abnormality, therefore attenuating the necessity of the ADAR1-dsRNA response axis. Second, why do the involved dsRNA sensors vary? The variance may reflect cell type-specific differences in dsRNA thresholds for different sensors, which are at least determined by sensor expression and ligand availability (Chen and Hur, 2022). With the lowest dsRNA threshold, the dominant sensor is most prone to aberrant activation. Therefore, when ADAR1’s suppression is experimentally compromised, this dominant sensor largely mediates the resulting abnormalities, and the rescue effect is more pronounced when the sensor is simultaneously inhibited. Also, the downstream signaling may have opposing effects in different cellular contexts. For instance, the ADAR1-MDA5-IFN axis is critical in embryos to prevent autoinflammation (Hu et al., 2023), but may be dispensable for HSPC emergence, where short-term IFN signaling is actually beneficial (Demerdash et al., 2021). Third, why is the editing-dependent mechanism not consistently necessary? Editing activity and binding activity of ADAR1 disrupt ligand-sensor recognition and compete for ligand, respectively, which corresponds to aforementioned qualitative and quantitative models for dsRNA immunogenicity. We hypothesize that the binding-dependent mechanism is essential while the editing-dependent one is auxiliary, given that other RNA-binding proteins lacking deaminase activity, can also suppress dsRNA response (Elbarbary et al., 2013; Cottrell et al., 2024). Nevertheless, they may cooperate to maintain homeostasis more robustly in most cases, and editing-mechanism is particularly important for dsRNAs with high immunogenic potential.

Admittedly, our hypothesis can be falsified under specific conditions. If the dependence on the ADAR1-dsRNA response axis were shown to be uniform across cytogenetically diverse leukemia cells, or across normal cells engineered to harbor different cytogenetic abnormalities, the notion of cytogenetics-driven supplementary pathways would be challenged. Also, if on an *Adar1*-deficient background, different sensors exhibited similar responsiveness to dsRNA stimulation regardless of their basal levels and dsRNA features, the threshold-based concept of sensor dominance would be contradicted. Regarding our proposed hierarchy of editing and binding activities, it would be falsified if editing-dead ADAR1 mutants failed to lower—or at least failed to maintain—the direct output of dsRNA response (e.g., reporter signals showing dsRNA-sensor binding), whereas editing-competent ones succeeded. Slightly, our hierarchy would also be challenged if the editing domain is conserved across species and under positive selection. Collectively, further investigation on cytogenetics-associated heterogeneity, dsRNA immunogenicity and ADAR evolution would help clarify the context-dependent ADAR1-dsRNA response axis in normal hematopoiesis and leukemia.

### 5.1 The dsRNA response: linking A-to-I editing & ADAR and normal hematopoiesis

The underlying principle for development is that ADAR1 (p150)-mediated suppression of MDA5-MAVS-IFN signaling safeguards cell survival, impacts hematopoiesis and maintains organismal homeostasis in a ubiquitous and lifelong manner, with editing-dependent and/or editing-independent mechanisms. The importance of the axis has been validated in embryonic hematopoiesis and adult lymphopoiesis, whereas no direct evidence has been reported for adult HSPCs and myelopoiesis (Table 2).

**FIGURE 8:**
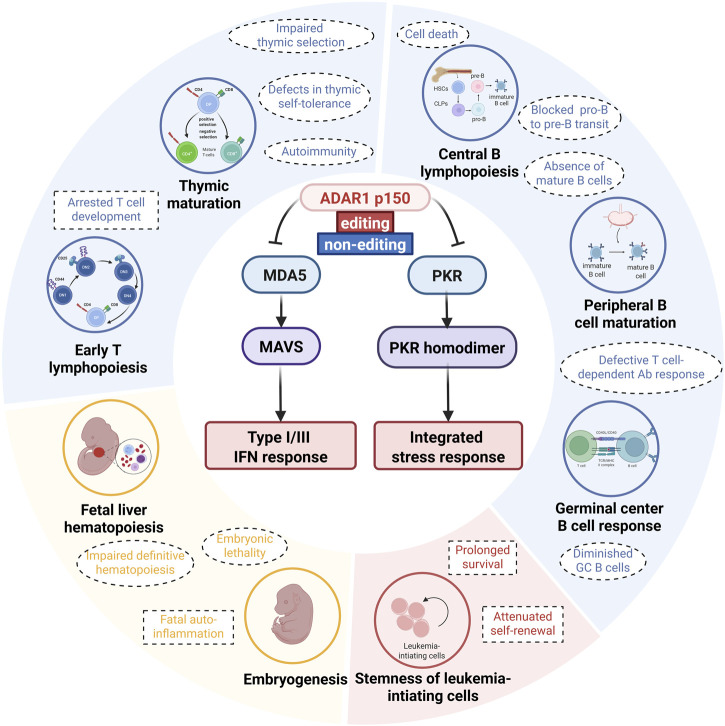
Current findings on how dsRNA response mediates the influence of ADAR1 and A-to-I editing on hematopoiesis and leukemia. The white dashed area corresponds to phenotypes caused by impairment of ADAR1 p150’s regulation on dsRNA response. Square boxes: phenotypes driven by both MDA5 and PKR pathways. Oval boxes: phenotypes driven exclusively by MDA5 pathway. Created with www.biorender.com.

The (at least partial) rescued phenotype by concurrent gene deletion provides compelling evidence that *Adar1* deficiency is tolerant in the absence of MDA5 sensing during embryogenesis (Mannion et al., 2014; Pestal et al., 2015; Heraud-Farlow et al., 2017; Hu et al., 2023), T lymphopoiesis (Nakahama et al., 2018; Vongpipatana et al., 2020), B lymphopoiesis (Chen et al., 2022; Li et al., 2022) and autoinflammatory pathogenesis (Nakahama et al., 2018; Liang et al., 2023a) (Figure 8). Notably, ADAR1’s immunosuppression in lymphopoiesis is essential for both early development and later maturation and functionality including thymic self-tolerance, T cell-mediated autoimmunity (Nakahama et al., 2018) and germinal center B cell response (Li et al., 2022). The weight of ADAR1-MDA5-MAVS axis shifts under different conditions, given that cell-specific *Adar1* and *Ifih1* co-deletion prevented spontaneous colitis successfully (Nakahama et al., 2018) whereas it only partially restored lymphocyte development (Vongpipatana et al., 2020; Chen et al., 2022) and B cell activation (Li et al., 2022). Therefore, ADAR1’s regulation on MDA5 pathway is necessary but insufficient for hematopoiesis in a cell-specific and context-dependent pattern, suggesting the involvement of additional mechanisms (see below).

Other findings imply the intimate relationship between ADAR1 p150 and IFN signaling, including 1) the predominant ADAR1 p150 expression in HSCs (Hartner et al., 2009; Liddicoat et al., 2016), granulocytes (Liddicoat et al., 2016) and erythrocytes (Liddicoat et al., 2016); 2) the stage-dependent changes of ADAR1 level during thymic T cell maturation (Nakahama et al., 2018; Vongpipatana et al., 2020); 3) the upregulated ISGs upon *Adar1* deletion in HSCs (Hartner et al., 2009), erythroid cells (Hartner et al., 2009; Liddicoat et al., 2016), pro-B and pre-B cells (Marcu-Malina et al., 2016; Chen et al., 2022), activated B cells (Li et al., 2022) and thymocytes (Nakahama et al., 2018; Vongpipatana et al., 2020). Recent isoform-specific gene deletion model bolsters the developmental significance of ADAR1 p150-IFN signaling throughout life, given the high ISG signature in germline *Adar1 p150*-deficient embryos and in induced *Adar1 p150*-deficient adult BM (Liang et al., 2023b).

Although generally indispensable, the detailed functions of the ADAR1-MDA5 axis vary during hematopoiesis, perhaps depending on cell types and contexts. First, the requirement of ADAR1’s editing function is mediated by MDA5 during embryonic hematopoiesis (Liddicoat et al., 2015; Pestal et al., 2015; Liddicoat et al., 2016; Heraud-Farlow et al., 2017), whereas the canonical dsRBD is sufficient to suppress MDA5 sensing during adult B lymphopoiesis (Chen et al., 2022) and maturation (Li et al., 2022). Additionally, MDA5 is also involved in ZBD-associated immunopathology (Nakahama et al., 2021; Liang et al., 2023a). According to our “essential binding and auxiliary editing” hierarchy, this discrepancy may reflect distinct dsRNA repertories during embryogenesis which require additional editing mechanism. Second, ADAR1 coordinates development and homeostasis with both MDA5-dependent and MDA5-independent mechanisms. In the absence of ADAR1, embryonic lethality was entirely attributable to activated MDA5-MAVS signaling while postnatal mortality partially resulted from MDA5-independent pathways (Pestal et al., 2015). Actually, *Adar1*-deficient E11.5 embryos also showed expression changes in development-related and metabolism-related genes, besides MAVS-related immune genes (Pestal et al., 2015). In early B lymphopoiesis, different mechanisms appear to be temporally separable. The MDA5-dependent ones promoted cell survival during early pro-B to large pre-B cell transition whereas the MDA5-independent ones regulated pre-BCR expression during pre-B to small pre-B transition (Chen et al., 2022). Similarly, early T cell development relies on ADAR1’s dual mechanisms in both MDA5 sensing and TCR expression (Vongpipatana et al., 2020). Third, ADAR1 p150 dominates in MDA5 suppression though both isoforms function cooperatively and irredundantly. Isoform-specific gene deletion has revealed the indispensable role of ADAR1 p150 in embryogenesis and adult homeostasis (Ward et al., 2011; Hu et al., 2023; Liang et al., 2023b). Furthermore, Pestal et al. (2015) reported that ADAR1 p150 prevented autoimmunity through regulating MDA5 sensing while both isoforms were important for multi-organ development (Pestal et al., 2015). Interestingly, the dsRNA binding activity of ADAR1 p150 was exclusively responsible for MDA5 inhibition in central B lymphopoiesis (Chen et al., 2022) and peripheral B maturation (Li et al., 2022), which could not be replaced by ADAR1 p110, even with the same localization. Actually, such ADAR1 p150 dominance has also been reported in mouse brains: upon ADAR1 p110 and ADAR2 depletion, only 2% remaining editing events successfully prevented MDA5 activation (Kim et al., 2021). The significance of bulk editing events occurring within non-immunogenic dsRNAs remains unclear.

Regarding other dsRNA sensors, ADAR1-mediated inhibition of PKR and OAS/RNaseL pathways is dispensable for embryonic apoptosis (Wang et al., 2004), early B lymphopoiesis (Chen et al., 2022) and GC response (Li et al., 2022). Nevertheless, PKR upregulation was reported in *Adar1*-deficient thymocytes, along with ISG upregulation (Xufeng et al., 2020). It is reasonable that MDA5 can activate PKR by inducing IFN signaling upon *Adar1* deletion. However, IFN signaling was not required for PKR activation in some human cell types perhaps because of their high levels of sensors or RNA substrates (Hu et al., 2023). Furthermore, ADAR1 p150 suppresses PKR activation via dsRBDs whereas its inhibition on MDA5 relies on RNA editing. Both suppression is indispensable for ADAR1 p150’s role in averting fatal autoinflammation though it is still unclear whether the two dsRNA pathways have crosstalk (Hu et al., 2023). Interestingly, in human HSPCs, editing on miRNA target sites of *Eif2ak2* transcript can disrupt miRNA-mediated inhibition of PKR expression (Wu et al., 2023), which may lower the ISR threshold to protect cells from perturbations.

### 5.2 The dsRNA response: an elusive link between A-to-I editing & ADAR and leukemia

Although the ADAR1-dsRNA response axis has been extensively explored in solid cancers (Ishizuka et al., 2019; Guallar et al., 2020; Zhang et al., 2022; Lin et al., 2023; Yee et al., 2025), there has been only one study validating its role in T-ALL to date. As mentioned in “ALL” subsection, ADAR1 contributes to the stemness of T-ALL LICs by avoiding aberrant dsRNA sensing and suppressing ISG expression, thereby promoting leukemia relapse and developing therapeutic resistance (Figure 8). Similar to the isoform-specific and multi-mechanism pattern in normal hematopoiesis, IFN-inducible ADAR1 p150 dominates the immunosuppression in T-ALL LICs through editing and non-editing activities. Both MDA5 and PKR can mediate ADAR1’s role as an LIC stemness factor. As shown in other contexts, ADAR1 prevents dsRNAs from activating MDA5 through both editing-dependent and editing-independent mechanisms, whereas only the editing-independent mechanism is required to suppress PKR detection (Rivera et al., 2024).

Intriguingly, the rescue effects on cell viability were diverse upon *Adar1* and *Ifih1* co-knockdown in different T-ALL PDX models and cell lines, where a greater rescue effect was consistent with a higher MDA5 expression level rather than a higher ADAR1 isoform expression level. Further analysis showed that cells with high dependence on the ADAR1-MDA5 axis harbored high ISG signatures (Rivera et al., 2024). Actually, the diverse dependence of cancer cells on the axis has also been observed in solid tumors (Gannon et al., 2018; Liu et al., 2019; Kung et al., 2021). The prevailing view is that intrinsically chronic ISG expression, possibly induced by aberrant DNA-activated IFN production, upregulates multiple dsRNA effectors and predisposes certain cancer cells to dsRNA accumulation, thereby rendering them sensitive to ADAR1 loss. PKR signaling and IFN signaling are consistently reported to mediate ADAR1 depletion-induced lethality. Nevertheless, upon IFNβ treatment, ADAR1-insensitive lung cancer cells acquired sensitivity (Gannon et al., 2018) whereas insensitive breast cancer cells remained refractory to ADAR1 loss (Kung et al., 2021), challenging ISG signature as the origin of heterogeneous ADAR1 dependencies. Here, in T-ALL, however, such heterogeneity is not identical to that in solid tumor, as it reflects the different responsiveness to MDA5 loss rather than to ADAR1 loss. And it remains unclear whether low responsiveness would be reversed by artificially elevating ISG signatures, and further, whether cytogenetics or other factors drive ISG differences. Beyond intra-leukemia heterogeneity, inter-leukemia heterogeneity also merits investigation. Notably, it is ambiguous whether *Adar1* deficiency activates IFN signaling, given the contradictory results in different leukemia types (Jiang et al., 2013; Steinman et al., 2013; Gassner et al., 2021; Rivera et al., 2024), though IFN-induced ADAR1 p150 expression has been validated (Gassner et al., 2021; Jiang et al., 2021; Rivera et al., 2024). The discrepancy may reflect subtype-specific dependence on the ADAR1-IFN signaling axis in leukemia. The heterogeneity model of dependence on the ADAR1-dsRNA response axis will be expanded after determining whether the sensitivity to ADAR1 loss varies across leukemias and, if so, whether different properties of dsRNA response (e.g., different dsRNA sensor expression) mediates the diverse sensitivity.

Another fascinating finding is that in *Adar1*-deficient T-ALL cells with high dependence on the axis, the increased total dsRNA levels did not trigger apoptosis in IFNβ-untreated conditions, indicating that T-ALL cells could tolerate a certain level of unedited dsRNAs (Rivera et al., 2024). In line with the “quantity” model discussed earlier, this “dsRNA tolerance threshold” may contribute to the dependence on the ADAR1-dsRNA response axis. Further investigation is warranted to determine how cells establish the tolerance threshold theoretically and how to measure it methodically. Moreover, it is also compelling to explore whether the threshold is cell-specific and whether it can be dysregulated during leukemogenesis.

The study did not delve into other dsRNA sensors, limited to the observations on 1) the comparable PKR expression across T-ALL cells, and 2) the solely editing-independent mechanism of ADAR1’s suppression on PKR. Interestingly, upregulated PKR following *Ifih1* knockdown in IFNβ-treated *Adar1*-deficient cells suggests crosstalk across dsRNA sensing pathways (Rivera et al., 2024).

### 5.3 Clinical potential of the ADAR1-dsRNA response axis in leukemia

If the ADAR1-dsRNA response axis is illuminated in leukemias, it would open up novel clinical opportunities, ranging from therapeutic strategies, resistance biomarkers, to prognostic predictors.

The rationale for targeting this axis in cancer therapy is that derepressing or activating the dsRNA response can trigger cell death, both intrinsically by initiating tumor-suppressive programmes and extrinsically by shaping inflammatory microenvironment. This yields two targeting strategies: braking the suppressive arm by inhibiting ADAR1, or accelerating the response by directly enhancing its elements. The “braking” strategy holds translational potential given the anti-leukemia phenotypes in pre-clinical models upon genetically *Adar1* depletion (Steinman et al., 2013; Zipeto et al., 2016; Rivera et al., 2024) or chemically ADAR1 inhibition (Crews et al., 2023; Shi et al., 2025). As summarised by Rehwinkel and Mehdipour (2025), ADAR1 inhibition can be further refined to isoform-specific and domain-specific inhibition. The “accelerating” strategy has multiple targets, including increasing dsRNA burden, lowering dsRNA threshold (if it exists), elevating the dsRNA sensor level and augmenting downstream signaling. For instance, DNA-demethylating agents can increase dsRNA burden by inducing “viral mimicry”—a cell state with enhanced dsRNA formation and accumulation, at least partially derived from endogenous retroviral elements (Chiappinelli et al., 2015; Roulois et al., 2015). Reasonably, the two strategies can synergize to enhance anti-tumor effects, pointing to the potential of ADAR1 in combination therapy beyond a standalone target. Encouragingly, ADAR1 inhibition has been shown to help overcome resistance against hypomethylating agents in colorectal cancer (Mehdipour et al., 2020) and ovarian cancer (Gomez et al., 2022), and against immunotherapy in melanoma (Ishizuka et al., 2019). Nevertheless, it remains unclear whether the synergistic effect also exists in leukemia.

Moreover, longitudinal monitoring of MDA5 levels (or other proxies evaluating the dsRNA response) during therapy could potentially identify emerging resistance, as a decline in these markers might indicate loss of dependence on the axis.

Although the possibly heterogeneous dependence on the ADAR1-dsRNA response axis may compromise the efficacy of above therapies, this feature can be harnessed for personalized medicine, with specific biomarkers predicting therapeutic response. Taking T-ALL as an example, quantifying pre-treatment MDA5 expression could guide clinical decisions on axis-targeted therapies. Moreover, if the reversibility of low dependence were established, patients with low dependence might also benefit from such therapies when combined with additional dependence-regulating interventions.

Despite its translational potentials, the risk of targeting the axis should be cautioned. The ideal therapeutic scenario is to target specific molecules exclusively in malignant cells. Consequently, off-target adverse effects (AEs) may arise either from engaging unintended molecules or from involving normal cells. These off-target AEs can be mitigated by optimized genetic manipulation and rigorous inhibitor screening, and have not been reported in leukemia-associated pre-clinical models given the spared normal hematopoietic cells upon targeting ADAR1 (Zipeto et al., 2016; Crews et al., 2023). By posing the immune system in an imbalanced status, axis-targeted therapies may also provoke on-target AEs, such as ADAR1-associated immunopathology, systemic autoinflammation and hyper-autoimmunity. It would be prudent to investigate whether and how locally favorable immune activation could become systemically harmful.

Additionally, to scrutinize above clinical potentials in pre-clinical models, certain proxies should be measured to assess axis activity, such as ADAR1 p150 level, p150/p110 ratio, A-to-I editing level, sensor level, ISG expression (e.g., IFIT1, ISG15), ISR signatures (e.g., eIF2a phosphorylation) and so on.

## 6 Conclusion

ADARs and A-to-I editing play an important role in hematological homeostasis through various mechanisms, operating in an isoform-specific and context-dependent manner. From embryonic to adult hematopoiesis, it is continuously required to maintain the proliferation-differentiation-apoptosis balance of hematopoietic cells, exerting distinct effects on specific lineages and specific stages. In leukemia, it contributes to tumor pathogenesis, progression and treatment resistance by regulating cell cycle-related elements. As summarized in Table 2, ADAR1, the major functional protein, mediates above effects via both editing-dependent and editing-independent activities-largely through suppressing dsRNA sensing, with its inhibition of MDA5 signaling being the most extensively studied to date (ADAR2, also included in Table 2, primarily functions through editing-dependent mechanisms not directly linked to dsRNA sensing.). We highlight the context-dependence of the ADAR1-dsRNA response axis in normal hematopoiesis and leukemia, attempting to elucidate it from three layers: 1) the shift in axis requirement from normal to malignant states; 2) the involvement of distinct dsRNA sensor(s); 3) the relative hierarchy between editing-dependent mechanism and editing-independent mechanism.

A striking trade-off exists between immune homeostasis and immune defense/surveillance in ADAR1-mediated regulation of dsRNA sensing. Physiologically, the ADAR1-dsRNA response axis prevents self-dsRNA from triggering autoinflammation while preparing hematopoietic cells for possible challenges. In contrast, pathologically, this immunosuppressive function can be exploited by leukemia stem cells to shape pro-oncogenic environment. Moreover, the dependence on the axis may exhibit intra-leukemia and inter-leukemia heterogeneity. Therefore, research on the ADAR1-dsRNA response axis not only advances our understanding of epitranscriptional regulation, but also suggests novel clinical potentials, ranging from therapeutic strategies, resistance biomarkers, to prognostic predictors. Given its accessibility, plasticity and dynamics, the hematological system provides an ideal context to elucidate the role of ADAR1 and A-to-I editing from an immunological perspective.

Several gaps remain unaddressed. First, it is still unclear whether the ADAR1-dsRNA axis influences adult hematopoietic lineages except lymphoid lineage, and whether it contributes to the pathogenesis of leukemia types other than ALL. Even for the existing findings, further work is needed to determine whether ambiguities stems from technical variations or inherent biological heterogeneity, especially for mechanical interaction between ADAR1 and dsRNA sensors. Second, more advanced tools are required. Current studies adopt genetic manipulation as the major strategy for ADAR1 exploration. Such drastic approaches may exaggerate the biological significance of the ADAR1-dsRNA response axis. Moreover, they often fail to capture transitional states between physiological and pathological conditions, which renders the ADAR1-regulated immune balance in “challenged” situations unresolved. The function of the axis has been investigated using human samples, xenograft models, mouse models and *in vitro* cell cultures. Nevertheless, it is challenging to accurately capture a single biological process due to the temporal and spatial complexity of hematological system. The advent of high-throughput sequencing has enabled RNA editome profiling, providing spatiotemporal insights and guiding further research. But such predictive information still requires validation through wet-lab experiments. Finally, it remains mysterious how important the ADAR1-dsRNA response axis is in the hematological system, and why the importance varies in a cell type-specific and context-dependent manner. A reasonable interpretation of diverse levels of dependence on the ADAR1-dsRNA response axis will establish a solid theoretical foundation for axis-targeted therapy. Future work should strive to move beyond qualitative observations toward quantitative evaluation.

## References

[B1] Allegra D. Bilan V. Garding A. Dohner H. Stilgenbauer S. Kuchenbauer F. (2014). Defective DROSHA processing contributes to downregulation of MiR-15/-16 in chronic lymphocytic leukemia. Leukemia 28 (1), 98–107. 10.1038/leu.2013.246 23974981

[B2] Bass B. L. Weintraub H. (1987). A developmentally regulated activity that unwinds RNA duplexes. Cell 48 (4), 607–613. 10.1016/0092-8674(87)90239-x 2434241

[B3] Beghini A. Ripamonti C. B. Peterlongo P. Roversi G. Cairoli R. Morra E. (2000). RNA hyperediting and alternative splicing of hematopoietic cell phosphatase (PTPN6) gene in acute myeloid leukemia. Hum. Mol. Genet. 9 (15), 2297–2304. 10.1093/oxfordjournals.hmg.a018921 11001933

[B4] Bortoletto E. Rosani U. (2024). Bioinformatics for inosine: tools and approaches to trace this elusive RNA modification. Genes (Basel) 15 (8), 996. 10.3390/genes15080996 39202357 PMC11353476

[B5] Cai M. Liu X. Luo A. Yang X. Yan Y. Liu S. (2023). ADAR1 polymorphisms contribute to increased susceptibility in pediatric acute lymphoblastic leukemia. Ann. Hematol. 102 (9), 2483–2492. 10.1007/s00277-023-05285-4 37217676

[B6] Chan C. P. Jin D. Y. (2022). Cytoplasmic RNA sensors and their interplay with RNA-binding partners in innate antiviral response: theme and variations. RNA 28 (4), 449–477. 10.1261/rna.079016.121 35031583 PMC8925969

[B7] Chen Y. G. Hur S. (2022). Cellular origins of dsRNA, their recognition and consequences. Nat. Rev. Mol. Cell Biol. 23 (4), 286–301. 10.1038/s41580-021-00430-1 34815573 PMC8969093

[B8] Chen C. X. Cho D. S. Wang Q. Lai F. Carter K. C. Nishikura K. (2000). A third member of the RNA-specific adenosine deaminase gene family, ADAR3, contains both single- and double-stranded RNA binding domains. RNA 6 (5), 755–767. 10.1017/s1355838200000170 10836796 PMC1369955

[B9] Chen W. Li Y. Ruan G. X. Huang H. Zhang R. Wang J. (2022). Adenosine deaminase acting on RNA-1 is essential for early B lymphopoiesis. Cell Rep. 41 (8), 111687. 10.1016/j.celrep.2022.111687 36417848

[B10] Chiappinelli K. B. Strissel P. L. Desrichard A. Li H. Henke C. Akman B. (2015). Inhibiting DNA methylation causes an interferon response in cancer via dsRNA including endogenous retroviruses. Cell 162 (5), 974–986. 10.1016/j.cell.2015.07.011 26317466 PMC4556003

[B11] Cottrell K. A. Ryu S. Pierce J. R. Soto Torres L. Bohlin H. E. Schab A. M. (2024). Induction of viral mimicry upon loss of DHX9 and ADAR1 in breast cancer cells. Cancer Res. Commun. 4 (4), 986–1003. 10.1158/2767-9764.CRC-23-0488 38530197 PMC10993856

[B12] Crews L. A. Jiang Q. Zipeto M. A. Lazzari E. Court A. C. Ali S. (2015). An RNA editing fingerprint of cancer stem cell reprogramming. J. Transl. Med. 13, 52. 10.1186/s12967-014-0370-3 25889244 PMC4341880

[B13] Crews L. A. Ma W. Ladel L. Pham J. Balaian L. Steel S. K. (2023). Reversal of malignant ADAR1 splice isoform switching with Rebecsinib. Cell Stem Cell 30 (3), 250–263. 10.1016/j.stem.2023.01.008 36803553 PMC10134781

[B14] D’Addabbo P. Cohen-Fultheim R. Twersky I. Fonzino A. Silvestris D. A. Prakash A. (2025). REDIportal: toward an integrated view of the A-to-I editing. Nucleic Acids Res. 53 (D1), D233–D242. 10.1093/nar/gkae1083 39588754 PMC11701558

[B15] Danan-Gotthold M. Guyon C. Giraud M. Levanon E. Y. Abramson J. (2016). Extensive RNA editing and splicing increase immune self-representation diversity in medullary thymic epithelial cells. Genome Biol. 17 (1), 219. 10.1186/s13059-016-1079-9 27776542 PMC5078920

[B16] Datta R. Adamska J. Z. Bhate A. Li J. B. (2023). A-to-I RNA editing by ADAR and its therapeutic applications: from viral infections to cancer immunotherapy. Wiley Interdiscip. Rev. RNA 15 (1), e1817. 10.1002/wrna.1817 PMC10947335 37718249

[B17] de Reuver R. Maelfait J. (2024). Novel insights into double-stranded RNA-mediated immunopathology. Nat. Rev. Immunol. 24 (4), 235–249. 10.1038/s41577-023-00940-3 37752355

[B18] Demerdash Y. Kain B. Essers M. A. G. King K. Y. (2021). Yin and Yang: the dual effects of interferons on hematopoiesis. Exp. Hematol. 96, 1–12. 10.1016/j.exphem.2021.02.002 33571568 PMC8919039

[B19] Eisenberg E. Levanon E. Y. (2018). A-to-I RNA editing - immune protector and transcriptome diversifier. Nat. Rev. Genet. 19 (8), 473–490. 10.1038/s41576-018-0006-1 29692414

[B20] Elbarbary R. A. Li W. Tian B. Maquat L. E. (2013). STAU1 binding 3' UTR IRAlus complements nuclear retention to protect cells from PKR-mediated translational shutdown. Genes Dev. 27 (13), 1495–1510. 10.1101/gad.220962.113 23824540 PMC3713430

[B21] Essers M. A. Offner S. Blanco-Bose W. E. Waibler Z. Kalinke U. Duchosal M. A. (2009). IFNalpha activates dormant haematopoietic stem cells *in vivo* . Nature 458 (7240), 904–908. 10.1038/nature07815 19212321

[B22] Gannon H. S. Zou T. Kiessling M. K. Gao G. F. Cai D. Choi P. S. (2018). Identification of ADAR1 adenosine deaminase dependency in a subset of cancer cells. Nat. Commun. 9 (1), 5450. 10.1038/s41467-018-07824-4 30575730 PMC6303303

[B23] Gassner F. J. Zaborsky N. Feldbacher D. Greil R. Geisberger R. (2020). RNA editing alters miRNA function in chronic lymphocytic leukemia. Cancers (Basel) 12 (5), 1159. 10.3390/cancers12051159 32380696 PMC7280959

[B24] Gassner F. J. Zaborsky N. Buchumenski I. Levanon E. Y. Gatterbauer M. Schubert M. (2021). RNA editing contributes to epitranscriptome diversity in chronic lymphocytic leukemia. Leukemia 35 (4), 1053–1063. 10.1038/s41375-020-0995-6 32728184 PMC8024191

[B25] George C. X. Samuel C. E. (1999). Human RNA-specific adenosine deaminase ADAR1 transcripts possess alternative exon 1 structures that initiate from different promoters, one constitutively active and the other interferon inducible. Proc. Natl. Acad. Sci. U. S. A. 96 (8), 4621–4626. 10.1073/pnas.96.8.4621 10200312 PMC16382

[B26] Golub R. Cumano A. (2013). Embryonic hematopoiesis. Blood Cells Mol. Dis. 51 (4), 226–231. 10.1016/j.bcmd.2013.08.004 24041595

[B27] Gomez S. Cox O. L. Walker R. R. Rentia U. Hadley M. Arthofer E. (2022). Inhibiting DNA methylation and RNA editing upregulates immunogenic RNA to transform the tumor microenvironment and prolong survival in ovarian cancer. J. Immunother. Cancer 10 (11), e004974. 10.1136/jitc-2022-004974 36343976 PMC9644370

[B28] Goncharov A. O. Shender V. O. Kuznetsova K. G. Kliuchnikova A. A. Moshkovskii S. A. (2022). Interplay between A-to-I editing and splicing of RNA: a potential point of application for cancer therapy. Int. J. Mol. Sci. 23 (9), 5240. 10.3390/ijms23095240 35563631 PMC9105294

[B29] Guallar D. Fuentes-Iglesias A. Souto Y. Ameneiro C. Freire-Agulleiro O. Pardavila J. A. (2020). ADAR1-Dependent RNA editing promotes MET and iPSC reprogramming by alleviating ER stress. Cell Stem Cell 27 (2), 300–314 e311. 10.1016/j.stem.2020.04.016 32396862 PMC7415614

[B30] Guo M. Chan T. H. M. Zhou Q. An O. Li Y. Song Y. (2023). Core-binding factor fusion downregulation of ADAR2 RNA editing contributes to AML leukemogenesis. Blood 141 (25), 3078–3090. 10.1182/blood.2022015830 36796022

[B31] Hartner J. C. Schmittwolf C. Kispert A. Muller A. M. Higuchi M. Seeburg P. H. (2004). Liver disintegration in the mouse embryo caused by deficiency in the RNA-editing enzyme ADAR1. J. Biol. Chem. 279 (6), 4894–4902. 10.1074/jbc.M311347200 14615479

[B32] Hartner J. C. Walkley C. R. Lu J. Orkin S. H. (2009). ADAR1 is essential for the maintenance of hematopoiesis and suppression of interferon signaling. Nat. Immunol. 10 (1), 109–115. 10.1038/ni.1680 19060901 PMC2701568

[B33] Heraud-Farlow J. E. Chalk A. M. Linder S. E. Li Q. Taylor S. White J. M. (2017). Protein recoding by ADAR1-mediated RNA editing is not essential for normal development and homeostasis. Genome Biol. 18 (1), 166. 10.1186/s13059-017-1301-4 28874170 PMC5585977

[B34] Herbert A. Alfken J. Kim Y. G. Mian I. S. Nishikura K. Rich A. (1997). A Z-DNA binding domain present in the human editing enzyme, double-stranded RNA adenosine deaminase. Proc. Natl. Acad. Sci. U. S. A. 94 (16), 8421–8426. 10.1073/pnas.94.16.8421 9237992 PMC22942

[B35] Higuchi M. Maas S. Single F. N. Hartner J. Rozov A. Burnashev N. (2000). Point mutation in an AMPA receptor gene rescues lethality in mice deficient in the RNA-editing enzyme ADAR2. Nature 406 (6791), 78–81. 10.1038/35017558 10894545

[B36] Hu S. B. Li J. B. (2024). RNA editing and immune control: from mechanism to therapy. Curr. Opin. Genet. Dev. 86, 102195. 10.1016/j.gde.2024.102195 38643591 PMC11162905

[B37] Hu S. B. Heraud-Farlow J. Sun T. Liang Z. Goradia A. Taylor S. (2023). ADAR1p150 prevents MDA5 and PKR activation via distinct mechanisms to avert fatal autoinflammation. Mol. Cell 83 (21), 3869–3884 e3867. 10.1016/j.molcel.2023.09.018 37797622 PMC13289476

[B38] Huang W. Sun Y. M. Pan Q. Fang K. Chen X. T. Zeng Z. C. (2022). The snoRNA-like lncRNA LNC-SNO49AB drives leukemia by activating the RNA-editing enzyme ADAR1. Cell Discov. 8 (1), 117. 10.1038/s41421-022-00460-9 36316318 PMC9622897

[B39] Ishizuka J. J. Manguso R. T. Cheruiyot C. K. Bi K. Panda A. Iracheta-Vellve A. (2019). Loss of ADAR1 in tumours overcomes resistance to immune checkpoint blockade. Nature 565 (7737), 43–48. 10.1038/s41586-018-0768-9 30559380 PMC7241251

[B40] Jiang Q. Crews L. A. Barrett C. L. Chun H. J. Court A. C. Isquith J. M. (2013). ADAR1 promotes malignant progenitor reprogramming in chronic myeloid leukemia. Proc. Natl. Acad. Sci. U. S. A. 110 (3), 1041–1046. 10.1073/pnas.1213021110 23275297 PMC3549099

[B41] Jiang Q. Isquith J. Zipeto M. A. Diep R. H. Pham J. Delos Santos N. (2019). Hyper-editing of cell-cycle regulatory and tumor suppressor RNA promotes malignant progenitor propagation. Cancer Cell 35 (1), 81–94 e87. 10.1016/j.ccell.2018.11.017 30612940 PMC6333511

[B42] Jiang Q. Isquith J. Ladel L. Mark A. Holm F. Mason C. (2021). Inflammation-driven deaminase deregulation fuels human pre-leukemia stem cell evolution. Cell Rep. 34 (4), 108670. 10.1016/j.celrep.2020.108670 33503434 PMC8477897

[B43] Karagianni K. Bibi A. Made A. Acharya S. Parkkonen M. Barbalata T. (2024). Recommendations for detection, validation, and evaluation of RNA editing events in cardiovascular and neurological/neurodegenerative diseases. Mol. Ther. Nucleic Acids 35 (1), 102085. 10.1016/j.omtn.2023.102085 38192612 PMC10772297

[B44] Kawahara Y. Zinshteyn B. Chendrimada T. P. Shiekhattar R. Nishikura K. (2007). RNA editing of the microRNA-151 precursor blocks cleavage by the Dicer-TRBP complex. EMBO Rep. 8 (8), 763–769. 10.1038/sj.embor.7401011 17599088 PMC1978079

[B45] Kim J. I. Nakahama T. Yamasaki R. Costa Cruz P. H. Vongpipatana T. Inoue M. (2021). RNA editing at a limited number of sites is sufficient to prevent MDA5 activation in the mouse brain. PLoS Genet. 17 (5), e1009516. 10.1371/journal.pgen.1009516 33983932 PMC8118328

[B46] Kumar B. V. Connors T. J. Farber D. L. (2018). Human T cell development, localization, and function throughout life. Immunity 48 (2), 202–213. 10.1016/j.immuni.2018.01.007 29466753 PMC5826622

[B47] Kung C. P. Cottrell K. A. Ryu S. Bramel E. R. Kladney R. D. Bao E. A. (2021). Evaluating the therapeutic potential of ADAR1 inhibition for triple-negative breast cancer. Oncogene 40 (1), 189–202. 10.1038/s41388-020-01515-5 33110236 PMC7796950

[B48] Levanon E. Y. Cohen-Fultheim R. Eisenberg E. (2024). In search of critical dsRNA targets of ADAR1. Trends Genet. 40 (3), 250–259. 10.1016/j.tig.2023.12.002 38160061

[B49] Li Y. Banerjee S. Goldstein S. A. Dong B. Gaughan C. Rath S. (2017). Ribonuclease L mediates the cell-lethal phenotype of double-stranded RNA editing enzyme ADAR1 deficiency in a human cell line. Elife 6, e25687. 10.7554/eLife.25687 28362255 PMC5404912

[B50] Li Y. Ruan G. X. Chen W. Huang H. Zhang R. Wang J. (2022). RNA-editing enzyme ADAR1 p150 isoform is critical for germinal center B cell response. J. Immunol. 209 (6), 1071–1082. 10.4049/jimmunol.2200149 35977796

[B51] Liang Z. Chalk A. M. Taylor S. Goradia A. Heraud-Farlow J. E. Walkley C. R. (2023a). The phenotype of the most common human ADAR1p150 Zalpha mutation P193A in mice is partially penetrant. EMBO Rep. 24 (5), e55835. 10.15252/embr.202255835 36975179 PMC10157378

[B52] Liang Z. Goradia A. Walkley C. R. Heraud-Farlow J. E. (2023b). Generation of a new Adar1p150 (-/-) mouse demonstrates isoform-specific roles in embryonic development and adult homeostasis. RNA 29 (9), 1325–1338. 10.1261/rna.079509.122 37290963 PMC10573302

[B53] Liang Z. Walkley C. R. Heraud-Farlow J. E. (2024). A-to-I RNA editing and hematopoiesis. Exp. Hematol. 139, 104621. 10.1016/j.exphem.2024.104621 39187172

[B54] Liao Y. Jung S. H. Kim T. (2020). A-to-I RNA editing as a tuner of noncoding RNAs in cancer. Cancer Lett. 494, 88–93. 10.1016/j.canlet.2020.08.004 32822814

[B55] Liddicoat B. J. Piskol R. Chalk A. M. Ramaswami G. Higuchi M. Hartner J. C. (2015). RNA editing by ADAR1 prevents MDA5 sensing of endogenous dsRNA as nonself. Science 349 (6252), 1115–1120. 10.1126/science.aac7049 26275108 PMC5444807

[B56] Liddicoat B. J. Hartner J. C. Piskol R. Ramaswami G. Chalk A. M. Kingsley P. D. (2016). Adenosine-to-inosine RNA editing by ADAR1 is essential for normal murine erythropoiesis. Exp. Hematol. 44 (10), 947–963. 10.1016/j.exphem.2016.06.250 27373493 PMC5035604

[B57] Lin W. Luo Y. Wu J. Zhang H. Jin G. Guo C. (2023). Loss of ADAR1 in macrophages in combination with interferon gamma suppresses tumor growth by remodeling the tumor microenvironment. J. Immunother. Cancer 11 (11), e007402. 10.1136/jitc-2023-007402 37935565 PMC10649901

[B58] Liu H. Golji J. Brodeur L. K. Chung F. S. Chen J. T. deBeaumont R. S. (2019). Tumor-derived IFN triggers chronic pathway agonism and sensitivity to ADAR loss. Nat. Med. 25 (1), 95–102. 10.1038/s41591-018-0302-5 30559422

[B59] Ma C. H. Chong J. H. Guo Y. Zeng H. M. Liu S. Y. Xu L. L. (2011). Abnormal expression of ADAR1 isoforms in Chinese pediatric acute leukemias. Biochem. Biophys. Res. Commun. 406 (2), 245–251. 10.1016/j.bbrc.2011.02.025 21316340

[B60] Mannion N. M. Greenwood S. M. Young R. Cox S. Brindle J. Read D. (2014). The RNA-editing enzyme ADAR1 controls innate immune responses to RNA. Cell Rep. 9 (4), 1482–1494. 10.1016/j.celrep.2014.10.041 25456137 PMC4542304

[B61] Marcu-Malina V. Goldberg S. Vax E. Amariglio N. Goldstein I. Rechavi G. (2016). ADAR1 is vital for B cell lineage development in the mouse bone marrow. Oncotarget 7 (34), 54370–54379. 10.18632/oncotarget.11029 27494846 PMC5342348

[B62] Meduri E. Breeze C. Marando L. Richardson S. E. Huntly B. J. P. (2022). The RNA editing landscape in acute myeloid leukemia reveals associations with disease mutations and clinical outcome. iScience 25 (12), 105622. 10.1016/j.isci.2022.105622 36465109 PMC9713371

[B63] Mehdipour P. Marhon S. A. Ettayebi I. Chakravarthy A. Hosseini A. Wang Y. (2020). Epigenetic therapy induces transcription of inverted SINEs and ADAR1 dependency. Nature 588 (7836), 169–173. 10.1038/s41586-020-2844-1 33087935

[B64] Nakahama T. Kawahara Y. (2023). The RNA-editing enzyme ADAR1: a regulatory hub that tunes multiple dsRNA-sensing pathways. Int. Immunol. 35 (3), 123–133. 10.1093/intimm/dxac056 36469491

[B65] Nakahama T. Kato Y. Kim J. I. Vongpipatana T. Suzuki Y. Walkley C. R. (2018). ADAR1-mediated RNA editing is required for thymic self-tolerance and inhibition of autoimmunity. EMBO Rep. 19 (12), e46303. 10.15252/embr.201846303 30361393 PMC6280791

[B66] Nakahama T. Kato Y. Shibuya T. Inoue M. Kim J. I. Vongpipatana T. (2021). Mutations in the adenosine deaminase ADAR1 that prevent endogenous Z-RNA binding induce aicardi-goutieres-syndrome-like encephalopathy. Immunity 54 (9), 1976–1988 e1977. 10.1016/j.immuni.2021.08.022 34525338

[B67] Oakes E. Anderson A. Cohen-Gadol A. Hundley H. A. (2017). Adenosine deaminase that acts on RNA 3 (ADAR3) binding to glutamate receptor subunit B Pre-mRNA inhibits RNA editing in glioblastoma. J. Biol. Chem. 292 (10), 4326–4335. 10.1074/jbc.M117.779868 28167531 PMC5354488

[B68] Ota H. Sakurai M. Gupta R. Valente L. Wulff B. E. Ariyoshi K. (2013). ADAR1 forms a complex with Dicer to promote microRNA processing and RNA-induced gene silencing. Cell 153 (3), 575–589. 10.1016/j.cell.2013.03.024 23622242 PMC3651894

[B69] Pestal K. Funk C. C. Snyder J. M. Price N. D. Treuting P. M. Stetson D. B. (2015). Isoforms of RNA-editing enzyme ADAR1 independently control nucleic acid sensor MDA5-Driven autoimmunity and multi-organ development. Immunity 43 (5), 933–944. 10.1016/j.immuni.2015.11.001 26588779 PMC4654992

[B70] Pu S. Cheng T. Cheng H. (2025). Advances in RNA editing in hematopoiesis and associated malignancies. Blood 145 (21), 2424–2438. 10.1182/blood.2024027379 39869834

[B71] Quelen C. Eloit Y. Noirot C. Bousquet M. Brousset P. (2016). RNA editing in acute myeloid leukaemia with normal karyotype. Br. J. Haematol. 173 (5), 788–790. 10.1111/bjh.13631 26251186

[B72] Rebagliati M. R. Melton D. A. (1987). Antisense RNA injections in fertilized frog eggs reveal an RNA duplex unwinding activity. Cell 48 (4), 599–605. 10.1016/0092-8674(87)90238-8 2434240

[B73] Rehwinkel J. Mehdipour P. (2025). ADAR1: from basic mechanisms to inhibitors. Trends Cell Biol. 35 (1), 59–73. 10.1016/j.tcb.2024.06.006 39030076 PMC11718369

[B74] Rivera M. Zhang H. Pham J. Isquith J. Zhou Q. J. Balaian L. (2024). Malignant A-to-I RNA editing by ADAR1 drives T cell acute lymphoblastic leukemia relapse via attenuating dsRNA sensing. Cell Rep. 43 (2), 113704. 10.1016/j.celrep.2024.113704 38265938 PMC10962356

[B75] Rossetti C. Picardi E. Ye M. Camilli G. D'Erchia A. M. Cucina L. (2017). RNA editing signature during myeloid leukemia cell differentiation. Leukemia 31 (12), 2824–2832. 10.1038/leu.2017.134 28484266 PMC5729351

[B76] Roulois D. Loo Yau H. Singhania R. Wang Y. Danesh A. Shen S. Y. (2015). DNA-demethylating agents target colorectal cancer cells by inducing viral mimicry by endogenous transcripts. Cell 162 (5), 961–973. 10.1016/j.cell.2015.07.056 26317465 PMC4843502

[B77] Shi Z. Li J. Ding J. Zhang Y. Min W. Zhu Y. (2025). ADAR1 is required for acute myeloid leukemia cell survival by modulating post-transcriptional wnt signaling through impairing miRNA biogenesis. Leukemia 39 (3), 599–613. 10.1038/s41375-024-02500-7 39702795

[B78] Smoak C. N. Gardner E. N. Chua R. N. Cottrell K. A. (2025). ZYS-1 is not an ADAR1 inhibitor. RNA 31 (12), 1703–1711. 10.1261/rna.080721.125 40957655 PMC12621589

[B79] Song B. Shiromoto Y. Minakuchi M. Nishikura K. (2022). The role of RNA editing enzyme ADAR1 in human disease. Wiley Interdiscip. Rev. RNA 13 (1), e1665. 10.1002/wrna.1665 34105255 PMC8651834

[B80] Steinman R. A. Yang Q. Gasparetto M. Robinson L. J. Liu X. Lenzner D. E. (2013). Deletion of the RNA-editing enzyme ADAR1 causes regression of established chronic myelogenous leukemia in mice. Int. J. Cancer 132 (8), 1741–1750. 10.1002/ijc.27851 22987615 PMC3565035

[B81] Sun T. Li Q. Geisinger J. M. Hu S. B. Fan B. Su S. (2025). ADAR1 editing is necessary for only a small subset of cytosolic dsRNAs to evade MDA5-mediated autoimmunity. Nat. Genet. 57 (12), 3101–3111. 10.1038/s41588-025-02430-9 41339703 PMC12695637

[B82] Tan M. H. Li Q. Shanmugam R. Piskol R. Kohler J. Young A. N. (2017). Dynamic landscape and regulation of RNA editing in mammals. Nature 550 (7675), 249–254. 10.1038/nature24041 29022589 PMC5723435

[B83] Teoh P. J. Koh M. Y. Chng W. J. (2021). ADARs, RNA editing and more in hematological malignancies. Leukemia 35 (2), 346–359. 10.1038/s41375-020-01076-2 33139858

[B84] Vongpipatana T. Nakahama T. Shibuya T. Kato Y. Kawahara Y. (2020). ADAR1 regulates early T cell development via MDA5-Dependent and -Independent pathways. J. Immunol. 204 (8), 2156–2168. 10.4049/jimmunol.1900929 32169840

[B85] Wang Q. Khillan J. Gadue P. Nishikura K. (2000). Requirement of the RNA editing deaminase ADAR1 gene for embryonic erythropoiesis. Science 290 (5497), 1765–1768. 10.1126/science.290.5497.1765 11099415

[B86] Wang Q. Miyakoda M. Yang W. Khillan J. Stachura D. L. Weiss M. J. (2004). Stress-induced apoptosis associated with null mutation of ADAR1 RNA editing deaminase gene. J. Biol. Chem. 279 (6), 4952–4961. 10.1074/jbc.M310162200 14613934

[B87] Wang F. He J. Liu S. Gao A. Yang L. Sun G. (2021). A comprehensive RNA editome reveals that edited Azin1 partners with DDX1 to enable hematopoietic stem cell differentiation. Blood 138 (20), 1939–1952. 10.1182/blood.2021011314 34388251 PMC8602937

[B88] Ward S. V. George C. X. Welch M. J. Liou L. Y. Hahm B. Lewicki H. (2011). RNA editing enzyme adenosine deaminase is a restriction factor for controlling measles virus replication that also is required for embryogenesis. Proc. Natl. Acad. Sci. U. S. A. 108 (1), 331–336. 10.1073/pnas.1017241108 21173229 PMC3017198

[B89] Weng S. Yang X. Yu N. Wang P. C. Xiong S. Ruan H. (2023). Harnessing ADAR-mediated site-specific RNA editing in immune-related disease: prediction and therapeutic implications. Int. J. Mol. Sci. 25 (1), 351. 10.3390/ijms25010351 38203521 PMC10779106

[B90] Wu X. Dao Thi V. L. Huang Y. Billerbeck E. Saha D. Hoffmann H. H. (2018). Intrinsic immunity shapes viral resistance of stem cells. Cell 172 (3), 423–438 e425. 10.1016/j.cell.2017.11.018 29249360 PMC5786493

[B91] Wu Y. Hao S. Xu X. Dong G. Ouyang W. Liu C. (2023). A novel computational method enables RNA editome profiling during human hematopoiesis from scRNA-seq data. Sci. Rep. 13 (1), 10335. 10.1038/s41598-023-37325-4 37365211 PMC10293275

[B92] Xiao H. Cheng Q. Wu X. Tang Y. Liu J. Li X. (2019). ADAR1 may be involved in the proliferation of acute myeloid leukemia cells via regulation of the Wnt pathway. Cancer Manag. Res. 11, 8547–8555. 10.2147/CMAR.S210504 31572009 PMC6759212

[B93] Xie W. Yang J. Zhou N. Ding H. Zhou G. Wu S. (2022). Identification of microRNA editing sites in three subtypes of leukemia. Front. Mol. Biosci. 9, 1014288. 10.3389/fmolb.2022.1014288 36452459 PMC9702332

[B94] Xing Y. Nakahama T. Wu Y. Inoue M. Kim J. I. Todo H. (2023). RNA editing of AZIN1 coding sites is catalyzed by ADAR1 p150 after splicing. J. Biol. Chem. 299 (7), 104840. 10.1016/j.jbc.2023.104840 37209819 PMC10404624

[B95] XuFeng R. Boyer M. J. Shen H. Li Y. Yu H. Gao Y. (2009). ADAR1 is required for hematopoietic progenitor cell survival via RNA editing. Proc. Natl. Acad. Sci. U. S. A. 106 (42), 17763–17768. 10.1073/pnas.0903324106 19805087 PMC2764897

[B96] Xufeng R. Nie D. Yang Q. Wang W. Cheng T. Wang Q. (2020). RNA editing enzyme ADAR1 is required for early T cell development. Blood Sci. 2 (1), 27–32. 10.1097/BS9.0000000000000039 35399867 PMC8974940

[B97] Yang Y. Sakurai M. (2025). Advances in detection methods for A-to-I RNA editing. Wiley Interdiscip. Rev. RNA 16 (2), e70014. 10.1002/wrna.70014 40223708 PMC11995373

[B98] Yang W. Chendrimada T. P. Wang Q. Higuchi M. Seeburg P. H. Shiekhattar R. (2006). Modulation of microRNA processing and expression through RNA editing by ADAR deaminases. Nat. Struct. Mol. Biol. 13 (1), 13–21. 10.1038/nsmb1041 16369484 PMC2950615

[B99] Yang Y. Okada S. Sakurai M. (2021). Adenosine-to-inosine RNA editing in neurological development and disease. RNA Biol. 18 (7), 999–1013. 10.1080/15476286.2020.1867797 33393416 PMC8216190

[B100] Yee P. S. Chai A. W. Y. Yee S. M. Ooi S. Tan Y. H. Garnett M. J. (2025). Interferon-inducible ADAR1 p150 is essential for the survival of oral squamous cell carcinoma. Mol. Carcinog. 64 (6), 1066–1077. 10.1002/mc.23910 40135601

[B101] Zhang T. Yin C. Fedorov A. Qiao L. Bao H. Beknazarov N. (2022). ADAR1 masks the cancer immunotherapeutic promise of ZBP1-driven necroptosis. Nature 606 (7914), 594–602. 10.1038/s41586-022-04753-7 35614224 PMC9373927

[B102] Zhang D. Zhu L. Gao Y. Wang Y. Li P. (2024). RNA editing enzymes: structure, biological functions and applications. Cell Biosci. 14 (1), 34. 10.1186/s13578-024-01216-6 38493171 PMC10944622

[B103] Zhu T. Niu G. Zhang Y. Chen M. Li C. Y. Hao L. (2023). Host-mediated RNA editing in viruses. Biol. Direct 18 (1), 12. 10.1186/s13062-023-00366-w 36978112 PMC10043548

[B104] Zipeto M. A. Court A. C. Sadarangani A. Delos Santos N. P. Balaian L. Chun H. J. (2016). ADAR1 activation drives leukemia stem cell self-renewal by impairing Let-7 biogenesis. Cell Stem Cell 19 (2), 177–191. 10.1016/j.stem.2016.05.004 27292188 PMC4975616

