# Supplementary material for: The context-dependent role of the dsRNA response in linking A-to-I editing and ADAR to normal hematopoiesis and leukemia
Source: Front Cell Dev Biol. 2026 May 25;14:1799320. doi: 10.3389/fcell.2026.1799320 (PMC13243261; doi:10.3389/fcell.2026.1799320)
Supplement: Supplementary file 1 [file Table1.docx]

***Supplementary Material***

1. **Supplementary Table**

**Table S1**. Current ADAR1-related *in vivo* animal experiments, findings and conclusions about embryonic hematopoiesis.

| Year | Model | Time | Survival | Phenotype | Concurrent Manipulation | Phenotype | Conclusions |
| --- | --- | --- | --- | --- | --- | --- | --- |
| 2000(1) | ADAR1^+/−^ chimerias | E13.5  E14.5 | Few  None | 1. Increased nucleated erythrocytes in PB 2. Unaffected number of hematopoietic cells in FL | NA | NA | ADAR1 is critical for embryonic erythropoiesis in the liver. |
| 2004(2) | Germline KO mice  (genotype: *Adar1*^+/-^)  (genotype: *Adar1^-^*^/-^) | P0  E8.5  E11.5  E12.0  E12.5 | All  All  All  Some  None | Normal primitive hematopoiesis  Decreased hematopoietic progenitors in YS, FL, PB | Chimera analysis | Undetectable contribution of *Adar1*^-/-^ cells to all hematopoietic tissues | ADAR1 is required for embryonic definitive hematopoiesis in a cell-autonomous fashion. |
| 2004(3) | Germline KO mice  (genotype: *Adar1*^+/-^)  (genotype: *Adar1^-^*^/-^) | P0  E9.5  E11.0  E12.5 | All  All  Some  None | Unaffected number of definitive hematopoietic progenitors in YS   1. Normal morphology of primitive erythrocytes 2. Increased apoptosis, esp. in FL | *Adar1* & *Eif2ak2* deletion | Embryonic lethality | ADAR1 is required for embryonic definitive hematopoiesis possibly by maintaining FL microenvironment. |
|  | Liver-specific KO mice  (genotype: *Alb*^Cre^*Adar1*^fl/fl^) | P0 | All | Hepatocellular damage but normal hematopoiesis | NA | NA |  |
| 2009(4) | Germline KO mice  (genotype: *Adar1^-^*^/-^) | E11.25 | All | Increased frequency but decreased number of HSC-enriched populations in FL | NA | NA | ADAR1 is dispensable for the emergence and migration of phenotypic HSCs in FL. |
|  | Inducible KO mice  (genotype: Mx1^Cre^Adar^f/-^) | P0 | All | Early death after birth | FL hematopoietic cell transplantation | Decreased contribution to hematopoiesis | ADAR1 is required in FL–derived HSCs in a cell-autonomous pattern. |
| 2015(5) | Germline MUT mice  (genotype: *Adar1*^E861A/+^)  (genotype: *Adar1*^E861A/E861A^) | P0  E12.5  E13.5  E14.5 | All  All  Some  None | 1. Decreased A-to-I editing frequency in erythroid-specific transcripts 2. Upregulated dsRNA response-related genes and ISGs in FL 3. A severe loss of erythroblasts in FL 4. Increased cell death of various hematopoietic populations in FL | *Adar1* mut &*Ifih1* deletion | Survived, with normal erythropoiesis | ADAR1-mediated RNA editing is required for FL erythropoiesis by preventing MDA5 from dsRNA sensing. |
| 2016(6) | Erythroid specific KO mice  (genotype: *Epor-Cre*^KI/+^*Adar1*^fl/−^)  (genotype: *Epor-Cre*^KI/+^*Adar1*^fl/E861A^) | E14.5  E15.5 | Some  Some | Blocked erythroid differentiation   1. Increased cell death within R4 and R5 fractions 2. Upregulated ISGs and genes related to dsRNA sensing in R2 and R3 fractions   Similar to above | FL transplantation  NA | A strong selection against Adar1^-/-^ cells   1. Decreased erythroid progenitors in BM 2. Anemia in PB 3. Splenomegaly   NA | ADAR1-mediated RNA editing is essential for normal embryonic erythropoiesis.. |
|  | Myeloid specific KO mice  (genotype: *LysM-Cre*^KI/+^*Adar1*^fl/−^) | P0 | All | Normal PB parameters | NA | NA | ADAR1 is not required for homeostatic maintenance of myeloid cells. |
| 2016(7) | Inducible KO mice  (genotype: *Meox2*^Cre^*Adar*^fl/fl^)  (genotype: *Meox2*^Cre^*Adar p150*^fl/fl^) | E11.5  P0  P0 | All  None  None | 1. Upregulated MAVS-related immune genes 2. Changed expression of development and metabolism-related genes | *Adar* & *Mavs* deletion  *Adar* & *Ddx58* deletion  *Adar* & *Mavs* deletion | Survived  Embryonic lethality  Survived | ADAR1 p150 is required for embryogenesis by regulating MDA5 pathway. |
| 2017(8) | Germline MUT&KO mice  (genotype: *Adar1*^+/+^*Ifih1*^-/-^)  (genotype:*Adar1*^E861A/+^*Ifih1*^-/-^)  (genotype:*Adar1*^E861A/E861A^*Ifih*1^-/-^) | P0  P0  P0 | All  All  All | Reduced weaning weight | NA | NA | ADAR1-mediated RNA editing is dispensable for embryogenesis once MDA5 is inactivated. |
| 2023(9) | Germline KO mice  (genotype: *Adar1 p150^-^*^/-^) | E12.5  P0 | Some  None | 1. Increased erythrocyte death in FL 2. Increased defective R1 in FL 3. Upregulated ISGs | NA | NA | ADAR1 p150 is essential for embryonic hematopoiesis possibly by suppressing IFN signaling. |
| 2023(10) | Germline MUT mice  (genotype: *Adar1 ^P195A/P195A^*)  (genotype: *Adar1 ^P195A/E861A^*) | P0  P0 | Al  All | Reduced weaning weight  Normal weaning weight | NA  NA | NA  NA | ADAR1 P195A mutation is well tolerated in isolation or combined with deficient editing. |

**Table S2**. Current ADAR1-related animal experiments, findings and conclusions about adult HSPCs.

| Year | Model | Phenotype | Concurrent Manipulation | Phenotype | Conclusions |
| --- | --- | --- | --- | --- | --- |
| 2009(11) | *Adar1*^Δ/Δ^ BM cell transplantation | Failed hematopoietic reconstitution of *Adar1*^Δ/Δ^ cells | NA | NA | ADAR1 is required for HPC survival via RNA editing. |
|  | *Adar1*^Δ/Δ^ BM LKS^+^ cell transplantation | As above | NA | NA |  |
|  | *Adar1*^Δ/Δ^ BM LKS^+^ cell *in vitro* culture | Normal proliferative rates | NA | NA |  |
|  | *Adar1*^Δ/Δ^ BM LKS^-^ cell *in vitro* culture | Decreased proliferative rates, attenuated colony forming ability, and increased apoptosis | *Adar1 p150 WT* reintroduction  *Adar1 E861A* reintroduction | Partially restored  Unrestored |  |
| 2009(4) | Inducible SCL^+^ cell-specific KO mice  (genotype: *Scl-Cre-ER*^T^Adar^f/−^) | 1. A strong selection against *Adar1*^-/-^ hematopoietic cells 2. Increased LKS^+^ cell percentage, and decreased LKS^-^ cell percentage in BM 3. Decreased LT-HSCs and increased S-phase cells within ST-HSCs | NA | NA | ADAR1 is essential for HSCs in adult BM. |
| 2017(8) | Germline MUT&KO mice  (genotype: *Adar1*^+/+^*Ifih1*^-/-^)  (genotype: *Adar1*^E861A/+^*Ifih1*^-/-^)  (genotype: *Adar1*^E861A/E861A^*Ifih*1^-/-^) | Generalized normal hematopoiesis  As above  As above | NA | NA | ADAR1-mediated editing in not required for homeostatic hematopoiesis once MDA5 is inactivated. |
| 2021(12) | Germline MUT mice  (genotype: *Adar1*^W197A/W197A^) | Increased LKS^+^ cell percentage, and decreased LKS^-^ cell percentage in BM | NA | NA | ADAR1 p150-mediated Z-RNA recognition is required for early hematopoiesis in adult BM. |
| 2023(9) | Inducible isoform-specific KO mice  (genotype: R26-CreER *Adar1*^fl/p150−^) | Generalized failure in hematopoiesis:   1. Decreased CMPs, increased HPC-1 cells in BM 2. Comparable number of HSCs and MPPs in BM 3. Upregulated ISGs in BM | NA | NA | ADAR1p150 is essential for HSPC in adult BM. |

**Table S3**. Current ADAR1-related animal experiments, findings and conclusions about adult lymphopoiesis.

| Year | Model | Phenotype | Concurrent Manipulation | Phenotype | Conclusions |
| --- | --- | --- | --- | --- | --- |
| 2016(13) | B cell-specific KO mice  (genotype: *Cd19*^Cre^*Adar1*^fl/fl^) | 1. Attenuated differentiation of early B precursors, increased apoptosis in late B precursors and disrupted final-stage B cell development in BM 2. Upregulated ISGs in late B precursors 3. Failed B cell maturation and maintenance in PB | NA | NA | ADAR1 is required for B cell development in BM and peripheral B cell maintenance. |
| 2018(14) | T cell-specific KO mice  (genotype: *Cd4*^Cre^*Adar1*^lox/lox^) | 1. Disrupted transition from DP to 4SP cells 2. Decreased RNA editing frequency, upregualted ISGs and attenuated TCRβ signaling in 4SP thymocytes 3. Dysregulated autoimmune response | *Adar1*& *Ifih1* deletion | Restored | ADAR1 is essential for thymocyte selection and thymic self-tolerance, possibly by inhibiting ISG expression. |
| 2020(15) | T cell-specific KO mice  (genotype: *Lck*^Cre^*Adar1*^lox/lox^ ) | 1. Disrupted differentiation at DN4 stage 2. Reduced number of peripheral T lymphocytes 3. Upregulated ISGs in thymocytes | NA | NA | ADAR1 is required for DN4 stage during thymocyte differentiation, possibly mediated by IFN pathway. |
| 2020(16) | Early T cell-specific KO mice  (genotype: *Lck*^Cre^*Adar1*^fl/fl^) | 1. Arrested thymocyte development at DN4 stage 2. Decreased thymocytes and mTECs in Thy 3. Decreased T cells in Sp 4. Increased apoptosis, increased ISG expression, decreased TCRβ expression and impaired TCRβ signaling in DN thymocytes | *Adar1* & *Ifih1* deletion  TCR transgene expression  Above deletion & expression | Partially restored  Partially restored  Restored | ADAR1 regulates early T Cell development through MDA5-dependent and -independent pathways, with the former affecting ISG expression and apoptosis, and the latter affecting TCRβ signaling and thymic maintenance. |
|  | Early T cell-specific MUT mice  (genotype: *Lck*^Cre^*Adar1*^E861A/E861A^) | NA | *Adar1* mutation& *Ifih1* deletion | Normal |  |
|  | Early T cell-specific KO mice  (genotype: *Lck*^Cre^*Adar2^f^*^l/fl^ ) | Normal | NA | NA |  |
| 2022(17) | Activated B cell-specific KO mice  (genotype: *Aicda*^Cre^*Adar1*^fl/fl^) | 1. Decreased germinal center B cells and decreased memory B cells 2. Reduced post-immunization IgG production 3. Hyperactivated IFN response in B cells | *Adar1* & *Ifih1* deletion  *Adar1* & *Eif2ak2* deletion  *Adar1* & *Rnasel* deletion  *Adar1 p150 WT* knockin  *Adar1 p110 WT* knockin  *Adar1 NES-p110* knockin  *Adar1 p150 E861A* knockin | Partially restored  Unrestored  Unrestored  Restored  Unrestored  Unrestored  Restored | ADAR1 p150 is required for the germinal center B response and T cell-dependent antibody response through binding to dsRNA and thereby suppressing MDA5 activation. |
| 2022(18) | B cell-specific KO mice  (genotype: *Mb1*^Cre^*Adar1*^fl/fl^)  *In vitro* BM cell culture  (genotype: *Mb1*^Cre^*Adar1*^fl/fl^) | 1. Blocked B lymphopoiesis at late pro-B stage 2. Impaired early-to-late pro-B differentiation 3. Severe B lymphopenia in PB 4. Increased apoptosis, decreased pre-BCR expression and type I IFN pathway hyperactivation of late pro-B and large pre-B cells   Decreased Fr. C and C’ B cells | *Adar1* & *Ifih1* deletion  *Adar1* & *Eif2ak2* deletion  *Adar1* & *Rnasel* deletion  *Adar1 p150 WT* knockin  *Adar1 p110 WT* knockin  *Adar1 p150 WT* expression  *Adar1 p150 P195A* expression  *Adar1 p150 E861A* expression | Partially restored  Unrestored  Unrestored  Restored  Unrestored  Restored  Restored  Restored | ADAR1 p150’s dsRNA-binding activity is essential for early B cell development in BM through both MDA5-dependent and -independent pathways. |
|  | B cell-specific KO&KI mice  (genotype: *Mb1*^Cre^*Adar1*^fl/fl^*MD4*^Tg^) | 1. Decreased mature B cells in BM 2. Decreased IgM expresion in immature B cells |  |  | ADAR1 is essential for immature B cell development. |
| 2023(9) | Inducible isoform-specific KO mice  (genotype: *R26-CreER*^T2^ *Adar1*^fl/p150-^) | Generalized failure in hematopoiesis:   1. Decreased B cells in PB and Sp 2. Decreased CD4+/CD8+ T ratio in Thy | NA | NA | ADAR1 p150 is required for adult hematopoiesis. |
| 2023(10) | Inducible non-selective KO mice  (genotype:*R26‐CreER*^T2^ *Adar1*^fl/P195A^) | 1. Upregulated ISGs, but well-tolerated 2. Modest changes in hematopoiesis | NA | NA | Impaired ADAR1 Zα domain activates well-tolerated IFN response. |

1. Wang Q, Khillan J, Gadue P, Nishikura K. Requirement of the RNA editing deaminase ADAR1 gene for embryonic erythropoiesis. Science. 2000;290(5497):1765-8.

2. Hartner JC, Schmittwolf C, Kispert A, Muller AM, Higuchi M, Seeburg PH. Liver disintegration in the mouse embryo caused by deficiency in the RNA-editing enzyme ADAR1. J Biol Chem. 2004;279(6):4894-902.

3. Wang Q, Miyakoda M, Yang W, Khillan J, Stachura DL, Weiss MJ, et al. Stress-induced apoptosis associated with null mutation of ADAR1 RNA editing deaminase gene. J Biol Chem. 2004;279(6):4952-61.

4. Hartner JC, Walkley CR, Lu J, Orkin SH. ADAR1 is essential for the maintenance of hematopoiesis and suppression of interferon signaling. Nat Immunol. 2009;10(1):109-15.

5. Liddicoat BJ, Piskol R, Chalk AM, Ramaswami G, Higuchi M, Hartner JC, et al. RNA editing by ADAR1 prevents MDA5 sensing of endogenous dsRNA as nonself. Science. 2015;349(6252):1115-20.

6. Liddicoat BJ, Hartner JC, Piskol R, Ramaswami G, Chalk AM, Kingsley PD, et al. Adenosine-to-inosine RNA editing by ADAR1 is essential for normal murine erythropoiesis. Exp Hematol. 2016;44(10):947-63.

7. Pestal K, Funk CC, Snyder JM, Price ND, Treuting PM, Stetson DB. Isoforms of RNA-Editing Enzyme ADAR1 Independently Control Nucleic Acid Sensor MDA5-Driven Autoimmunity and Multi-organ Development. Immunity. 2015;43(5):933-44.

8. Heraud-Farlow JE, Chalk AM, Linder SE, Li Q, Taylor S, White JM, et al. Protein recoding by ADAR1-mediated RNA editing is not essential for normal development and homeostasis. Genome Biol. 2017;18(1):166.

9. Liang Z, Goradia A, Walkley CR, Heraud-Farlow JE. Generation of a new Adar1p150 (-/-) mouse demonstrates isoform-specific roles in embryonic development and adult homeostasis. RNA. 2023;29(9):1325-38.

10. Liang Z, Chalk AM, Taylor S, Goradia A, Heraud-Farlow JE, Walkley CR. The phenotype of the most common human ADAR1p150 Zalpha mutation P193A in mice is partially penetrant. EMBO Rep. 2023;24(5):e55835.

11. XuFeng R, Boyer MJ, Shen H, Li Y, Yu H, Gao Y, et al. ADAR1 is required for hematopoietic progenitor cell survival via RNA editing. Proc Natl Acad Sci U S A. 2009;106(42):17763-8.

12. Nakahama T, Kato Y, Shibuya T, Inoue M, Kim JI, Vongpipatana T, et al. Mutations in the adenosine deaminase ADAR1 that prevent endogenous Z-RNA binding induce Aicardi-Goutieres-syndrome-like encephalopathy. Immunity. 2021;54(9):1976-88 e7.

13. Marcu-Malina V, Goldberg S, Vax E, Amariglio N, Goldstein I, Rechavi G. ADAR1 is vital for B cell lineage development in the mouse bone marrow. Oncotarget. 2016;7(34):54370-9.

14. Nakahama T, Kato Y, Kim JI, Vongpipatana T, Suzuki Y, Walkley CR, et al. ADAR1-mediated RNA editing is required for thymic self-tolerance and inhibition of autoimmunity. EMBO Rep. 2018;19(12).

15. Xufeng R, Nie D, Yang Q, Wang W, Cheng T, Wang Q. RNA editing enzyme ADAR1 is required for early T cell development. Blood Sci. 2020;2(1):27-32.

16. Vongpipatana T, Nakahama T, Shibuya T, Kato Y, Kawahara Y. ADAR1 Regulates Early T Cell Development via MDA5-Dependent and -Independent Pathways. J Immunol. 2020;204(8):2156-68.

17. Li Y, Ruan GX, Chen W, Huang H, Zhang R, Wang J, et al. RNA-Editing Enzyme ADAR1 p150 Isoform Is Critical for Germinal Center B Cell Response. J Immunol. 2022;209(6):1071-82.

18. Chen W, Li Y, Ruan GX, Huang H, Zhang R, Wang J, et al. Adenosine deaminase acting on RNA-1 is essential for early B lymphopoiesis. Cell Rep. 2022;41(8):111687.
